# Switching the proton-coupled electron transfer mechanism for non-canonical tyrosine residues in a *de novo* protein†

**DOI:** 10.1039/d3sc05450k

**Published:** 2024-01-25

**Authors:** Astrid Nilsen-Moe, Clorice R. Reinhardt, Ping Huang, Hemlata Agarwala, Rosana Lopes, Mauricio Lasagna, Starla Glover, Sharon Hammes-Schiffer, Cecilia Tommos, Leif Hammarström

**Affiliations:** a Department of Chemistry, Ångström Laboratory, Uppsala University Box 523 75120 Uppsala Sweden leif.hammarstrom@kemi.uu.se; b Department of Molecular Biophysics and Biochemistry, Yale University New Haven CT 06520 USA; c Technical University Munich, Campus Straubing for Biotechnology and Sustainability Uferstraße 53 94315 Straubing Germany; d Department of Biochemistry and Biophysics, Texas A&M University College Station TX 77843 USA tommos@tamu.edu; e Department of Chemistry, Yale University New Haven CT 06520 USA

## Abstract

The proton-coupled electron transfer (PCET) reactions of tyrosine (Y) are instrumental to many redox reactions in nature. This study investigates how the local environment and the thermodynamic properties of Y influence its PCET characteristics. Herein, 2- and 4-mercaptophenol (MP) are placed in the well-folded α_3_C protein (forming 2MP-α_3_C and 4MP-α_3_C) and oxidized by external light-generated [Ru(L)_3_]^3+^ complexes. The resulting neutral radicals are long-lived (>100 s) with distinct optical and EPR spectra. Calculated spin-density distributions are similar to canonical Y˙ and display very little spin on the S–S bridge that ligates the MPs to C_32_ inside the protein. With 2MP-α_3_C and 4MP-α_3_C we probe how proton transfer (PT) affects the PCET rate constants and mechanisms by varying the degree of solvent exposure or the potential to form an internal hydrogen bond. Solution NMR ensemble structures confirmed our intended design by displaying a major difference in the phenol OH solvent accessible surface area (≤∼2% for 2MP and 30–40% for 4MP). Additionally, 2MP-C_32_ is within hydrogen bonding distance to a nearby glutamate (average O–O distance is 3.2 ± 0.5 Å), which is suggested also by quantum mechanical/molecular mechanical (QM/MM) molecular dynamics simulations. Neither increased exposure of the phenol OH to solvent (buffered water), nor the internal hydrogen bond, was found to significantly affect the PCET rates. However, the lower phenol p*K*_a_ values associated with the MP-α_3_C proteins compared to α_3_Y provided a sufficient change in PT driving force to alter the PCET mechanism. The PCET mechanism for 2MP-α_3_C and 4MP-α_3_C with moderately strong oxidants was predominantly step-wise PTET for pH values, but changed to concerted PCET at neutral pH values and below when a stronger oxidant was used, as found previously for α_3_Y. This shows how the balance of ET and PT driving forces is critical for controlling PCET mechanisms. The presented results improve our general understanding of amino-acid based PCET in enzymes.

## Abstract 2

Proton-coupled electron transfer reactions of tyrosine are instrumental to many redox reactions in nature. By balancing the driving forces for electron and proton transfer, the mechanism can be changed from step-wise to concerted transfer (CEPT).

## Graphical abstract

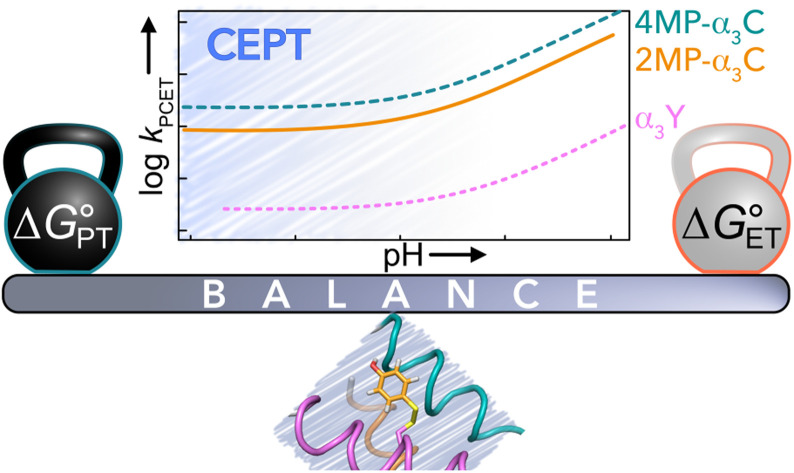

## Introduction

Proton-coupled electron transfer (PCET) is a fundamental process that is ubiquitous in natural and synthetic redox chemistry and catalysis. Understanding how PCET functions in biochemical systems unlocks the potential to take advantage of the same basic principles in synthetic designs. PCET can proceed *via* a step-wise mechanism where electron transfer (ET) and proton transfer (PT) advance one after the other (PTET or ETPT), or *via* a concerted mechanism (CEPT) where both ET and PT proceed in one kinetic step. Theoretical and small-molecule experimental studies have shown that PCET rate constants and mechanisms depend on the driving forces for electron and proton transfer, 
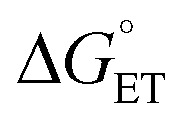
 and 
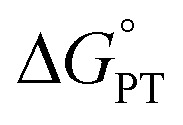
, electron and proton transfer (tunneling) distances, and the reorganization energy, *λ*.^1–4^ Systematic studies that examine how PCET is affected by changing 
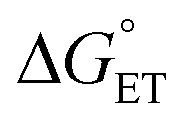
 and 
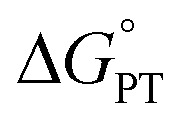
 are important to advance our understanding of PCET in biology and chemistry.

Some oxidoreductases use tyrosine (Y), tryptophan (W), glycine, and/or cysteine residues as 1e^−^ redox (radical) cofactors.^3,5^ Amino-acid oxidation–reduction typically involves PCET, with the exception of W which participates in both 1e^−^ and 1e^−^/1H^+^ reactions. In the context of PCET, 
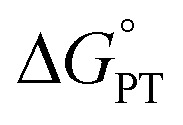
 is determined by the p*K*_a_ of the amino acid and the p*K*_a_ of the primary proton acceptor. The latter may be a protein residue, a cofactor, buffer and/or water species. Modulating the 
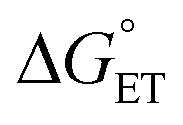
 and/or 
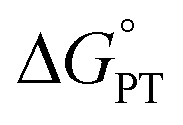
 parameter(s) can change the mechanism between step-wise and concerted PCET.^1^ This has important consequences for the rate of radical generation and transfer. If PCET is a part of the rate-limiting step in a catalytic cycle, changes in the mechanism can consequently affect catalytic behavior and performance.

Here we use the α_3_X protein model system to alter the local environment of a Y redox site and investigate if, and in that case how, the structural changes alter the PCET properties. The α_3_X family of well-structured model proteins is based on a 65-residue, pH stable and redox inert three-helix bundle (α_3_).^5,6^ The α_3_ scaffold hosts a single redox-active Y or W residue at interior position 32 (X_32_). Oxidation-reduction of X_32_ is reversible, allowing accurate midpoint potentials, *E*°′, (*i.e.*, [radical]/[reduced species] = 1) to be obtained.^7,8^ This represents a major advantage of using the α_3_X model system to characterize amino-acid based PCET reactions. Additionally, the broad pH stability of the α_3_X proteins allows PCET characterization as a function of pH. Tommos *et al.* introduced a series of noncanonical Y residues at site 32, including aminotyrosine (α_3_(NH_2_)Y), fluorotyrosines (*α*_3_(F_*n*_)Y, *n* = 2, 3), and covalently bound mercaptophenols (2MP- and 4MP-α_3_C).^8–11^ With these Y analogs, the *E*°′(X_32_˙/X_32_) and the phenol p*K*_a_ could be expanded across a range of 722 mV and 4.1 p*K*_a_ units, respectively.^5^ In this study, we report the structural, spectroscopic, and radical (X_32_˙) formation and decay characteristics of 2MP-α_3_C and 4MP-α_3_C relative to those of α_3_Y. Previous studies using external [Ru(bpy)_3_]^3+^ (bpy = 2,2′-bipyridine) oxidants showed that the 1e^−^/1H^+^ oxidation of Y_32_ is pH-dependent with CEPT dominating at low pH and pre-equilibrium PTET dominating at high pH.^12,13^ Water (H_2_O) was assigned as the dominant primary proton acceptor for the CEPT mechanism. Y_32_˙ was shown to be long-lived (*t*_1/2_ = 2–10 s) and to decay *via* radical–radical dimerization.^12^

**Fig. 1:**
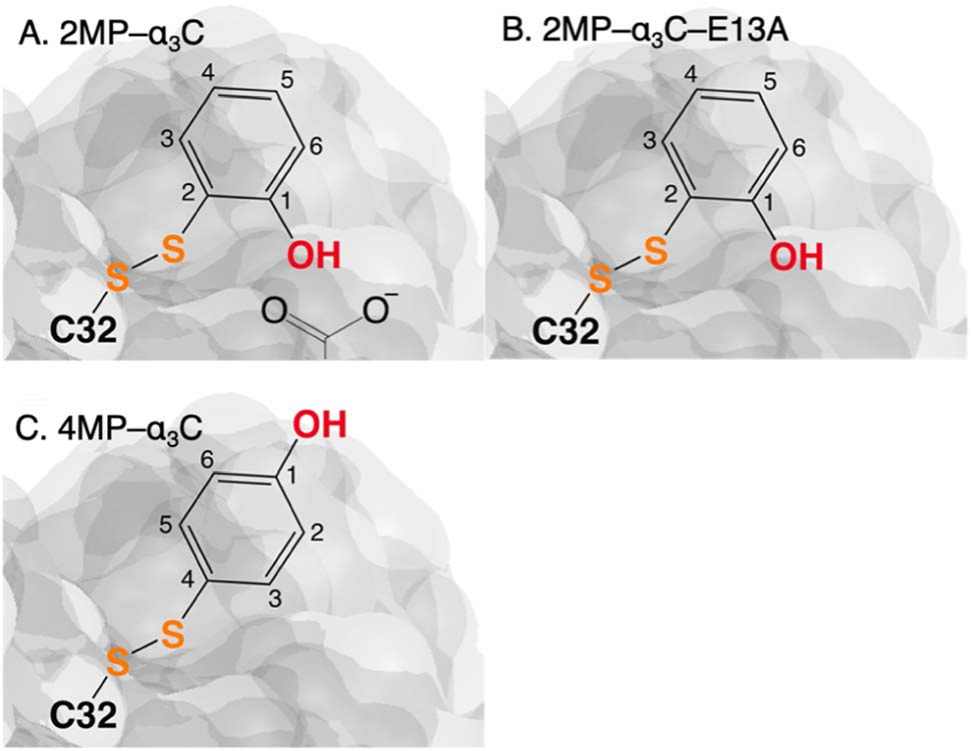
Schematic description illustrating the protein design and key differences between the MP-C_32_ site in (A) 2MP-α_3_C, (B) 2MP-α_3_C-E_13_A, and (C) 4MP-α_3_C. Modified with permission from ref. 14 Copyright © 2013 American Chemical Society.

The MP-α_3_C proteins were designed to specifically modulate interactions at the phenol OH group. By ligating the different MPs to the buried C_32_ residue, the aim was to shift the phenol OH from the protein interior (2MP-α_3_C, Fig. 1A) towards the protein surface (4MP-α_3_C, Fig. 1C).^9^ The solution nuclear magnetic resonance (NMR) structure of 2MP-α_3_C confirmed the intended design for this protein.^14^ This structure also revealed that 2MP-C_32_ is involved in a weak, interhelical hydrogen bond (H-bond) with the sidechain oxygen(s) of E_13_. The solution NMR structure of 4MP-α_3_C, presented herein, solidifies the protein design further by showing that the solvent accessible surface area (SASA) of the phenol OH changes from ≤∼2% in 2MP-α_3_C to 30–40% in 4MP-α_3_C.

Using transient absorption (TA) spectroscopy, we show the light-induced formation of 2MP˙-C_32_ and 4MP˙-C_32_ radicals, which are long-lived (*t*_1/2_ > 100 s) and exhibit different optical and EPR spectra. Calculations show the alternate spin-density distribution patterns typical of neutral (deprotonated) phenol radicals with only minor spin densities on the sulfur atoms. We found that the rate and mechanism by which X_32_ is oxidized are not sensitive to a major change in the phenol OH SASA nor removing the 2MP-C_32_/E_13_ interaction. Instead, we observed that a 1.6 unit decrease in the phenol p*K*_a_ compared to α_3_Y is sufficient to alter the oxidation reaction from a pH-dependent, mixed CEPT/PTET mechanism to mainly following a PTET pathway. Interestingly, this p*K*_a_ driven change in the X_32_ oxidation mechanism can be reversed by increasing the 
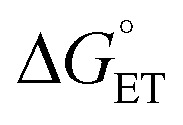
. Our results highlight the critical balance between ET and PT driving forces in controlling PCET mechanisms.

## Materials and methods

### Transient absorption sample preparation

2MP- and 4MP-α_3_C were prepared as described earlier^14^ and stored as lyophilized protein powder. Lyophilized protein was dissolved in 100 mM phosphate buffer KP_i_ (KH_2_PO_4_ from Sigma Life Science ≥99% purity, K_2_HPO_4_ from ACROS Organics 99%+ purity), containing 40 mM KCl (Alfa Aesar 99.0–100.5% purity). In experiments where rate constants were measured as a function of buffer concentration, the following concentrations were used: [KP_i_], 20–400 mM; [2MP-α_3_C], 320–360 μM; [4MP-α_3_C], 240 μM; [Ru(bpy)_3_]Cl_2_, 20–30 μM; and [Co(NH_3_)_5_Cl]Cl_2_, 3–5 mM. In experiments where rate constants were measured as a function of pH, the following concentrations were used: [KP_i_], 100 mM; [2MP-α_3_C,] 240–390 μM; [4MP-α_3_C], 170–540 μM; [2MP-α_3_C-E_13_A], 330 μM; [Ru(bpy)_3_]Cl_2_, 20-30 μM; [Ru(dmb)_3_]Cl_2_, 20–30 μM; [Ru(deeb)_3_]Cl_2_, 20–30 μM; [Co(NH_3_)_5_Cl]Cl_2_, 4–6 mM; and [Na_2_S_2_O_8_], 5 mM. Protein, photosensitizer and quencher concentrations were determined spectrophotometrically using a Cary 50 UV-vis spectrometer and extinction coefficients: *ε*_290_(2MP-α_3_C) 3700 M^−1^ cm^−1^;^9^*ε*_290_(4MP-α_3_C) 2300 M^−1^ cm^−1^;^9^*ε*_452_([Ru(bpy)_3_]^2+^) 14 600 M^−1^ cm^−1^;^15^*ε*_460_([Ru(dmb)_3_]^2+^) 14 600 M^−1^ cm^−1^;^15^*ε*_464_([Ru(deeb)_3_]^2+^) 23 300 M^−1^ cm^−1^;^15^*ε*_532_([Co(NH_3_)_5_Cl]^2+^) 52 M^−1^ cm^−1^.^12^ Photosensitizer and quencher solutions were always prepared separately and mixed under dark conditions. For the α_3_X samples, the protein was added to the photosensitizer solution prior to mixing with the quencher solution. The solution pH was adjusted with 0.1–1 M NaOH and 0.01–1 M HCl and measured using a calibrated Metrohm LL Biotrode pH-electrode.

### Transient absorption methods

The TA laser flash-photolysis setup has previously been described in detail.^12,16,17^ Briefly, the sample was excited using a Nd:YAG laser (Quantel, BrilliantB) with the laser light passed through an OPO tuned to 460 nm. Care was taken to avoid probe-light photochemistry during each experiment, and irreversible photoconversion of the sample by ambient, laser or probe light prior to the actual experiment. The probe light was first passed through a monochromator (Applied Photophysics, pbp Spectra Kinetic Monochromator 05-109 with slit widths set to 4 mm in and out) before hitting the sample at a 90° angle relative to the excitation light. After the sample, the probe light was passed through a 2^nd^ monochromator (same model as listed above with slit widths set to 2 mm in and out) before reaching the PMT detector (Hamamatsu R928). The signal was digitized in a digital oscilloscope (Agilent Technologies Infiniium 600 MHz). TA traces were produced with the Applied Photophysics LKS software package. The laser power was 10–13 mJ per shot. TA spectra were recorded on a UV-vis spectrometer (Agilent 8453 diode array). The sample was excited using a 447.5 nm LED (Luxeion Star, Rebel premounted LED fitted with carlco 29.8/10 mm lens) controlled by an HP 8116A 50 MHz pulse/function generator to supply a reproducible pulse length of 500 ms.

TA samples were contained in a 4 × 10 mm cuvette with an extra-long neck to avoid losing sample during deoxygenation. For the flash-photolysis measurements, the probe light was led through the 10 mm pathlength, and for the TA spectra, the probe light was led through the 4 mm pathlength. When [Co(NH_3_)_5_Cl]Cl_2_ was used as the quencher, oxygen was excluded from the sample by gently purging with high purity Ar gas for 10 minutes. When Na_2_S_2_O_8_ was used as the quencher, oxygen was not removed. All experiments were carried out at 23 (±1) °C.

Changes in pH of *ca.* 0.1–0.2 units were observed for flash-photolysis samples. The pH was therefore measured before and after TA, and the average values reported here.

### Solution NMR spectroscopy

^13^C,^15^N-α_3_C expression and purification, MP labeling, and NMR sample preparations were conducted as described previously.^14,18^ Standard multidimensional NMR experiments were conducted at 30 °C using a 750 MHz Bruker Avance III spectrometer equipped with a cryoprobe. ^1^H, ^13^C, and ^15^N resonance assignments were made as described in ref. 14 and 18. NOE-based distance restraints were obtained as described in ref. 18. NMR data were processed with Felix95 (Accelrys Inc., San Diego, CA) and analyzed with SPARKY.^19^ Structural calculations were performed with the CNS software suite,^20^ as described in ref. 12. SASA analyses were perform with MOLMOL.^21^ Structural coordinates (RCSB Protein Data Bank ID 8VSW) and NMR chemical shifts (Biological Magnetic Resonance Data Bank, BMRB ID 31067) have been deposited for 4MP-α_3_C.

### X-band EPR spectroscopy

All electron paramagnetic resonance spectra were recorded on a Bruker EMX-micro spectrometer equipped with an EMX-Primium bridge and an ER4119HS resonator. Individual solutions were deoxygenated before mixing and the final sample concentrations were 230–250 μM protein, 20–30 μM [Ru(bpy)_3_]^2+^, and 4.5 mM [Co(NH_3_)_5_Cl]^2+^. Each sample was ∼100 μL and contained in a flat cell. A dark spectrum was recorded before the sample was exposed to *in situ* continuous illumination of a 447.5 nm LED (same setup as above) at ambient atmosphere. EPR settings: microwave frequency, 9.85 GHz; microwave power 6.3 mW; modulation frequency 100 kHz; modulation amplitude 0.1 mT. The Xepr software package (Bruker) was used for data acquisition and processing.

### Computational studies

Geometry optimizations were performed using density functional theory (DFT) with Gaussian 16.^22^ The DFT calculations used the B3LYP-D3(BJ),^23,24^ ωB97X-D,^25^ and M06-2X^26^ density functionals and various basis sets as specified. In addition, complete active space self-consistent field (CASSCF) calculations were performed with the aug-cc-pVTZ basis set^27,28^ using the PySCF program^29,30^ for geometries optimized at the DFT ωB97X-D/6-31+G** level. The active spaces were chosen with the automated π-orbital space (PiOS) method,^31^ which constructed a (9e, 8o) active space for the 4MP and 2MP models and a (7e,7o) active space for the Y model. Mulliken spin population analyses were conducted for the various radical systems.

To investigate H-bonding interactions, classical molecular dynamics (MD) simulations of the 2MP-α_3_C and 4MP-α_3_C proteins were performed with Amber20 (ref. 32) using the ff14SB forcefield^33^ with TIP3P water.^34^ The simulation protocol was similar to our previous computational studies on α_3_Y proteins.^13^ Detailed H-bonding analyses were conducted for 1 microsecond trajectories. An additional 5 ps quantum mechanical/molecular mechanical (QM/MM) trajectory was also propagated. Complete computational details are provided in the ESI.†

## Results and discussion

### Site 32 in α_3_Y, 2MP-α_3_C and 4MP-α_3_C

**Fig. 2:**
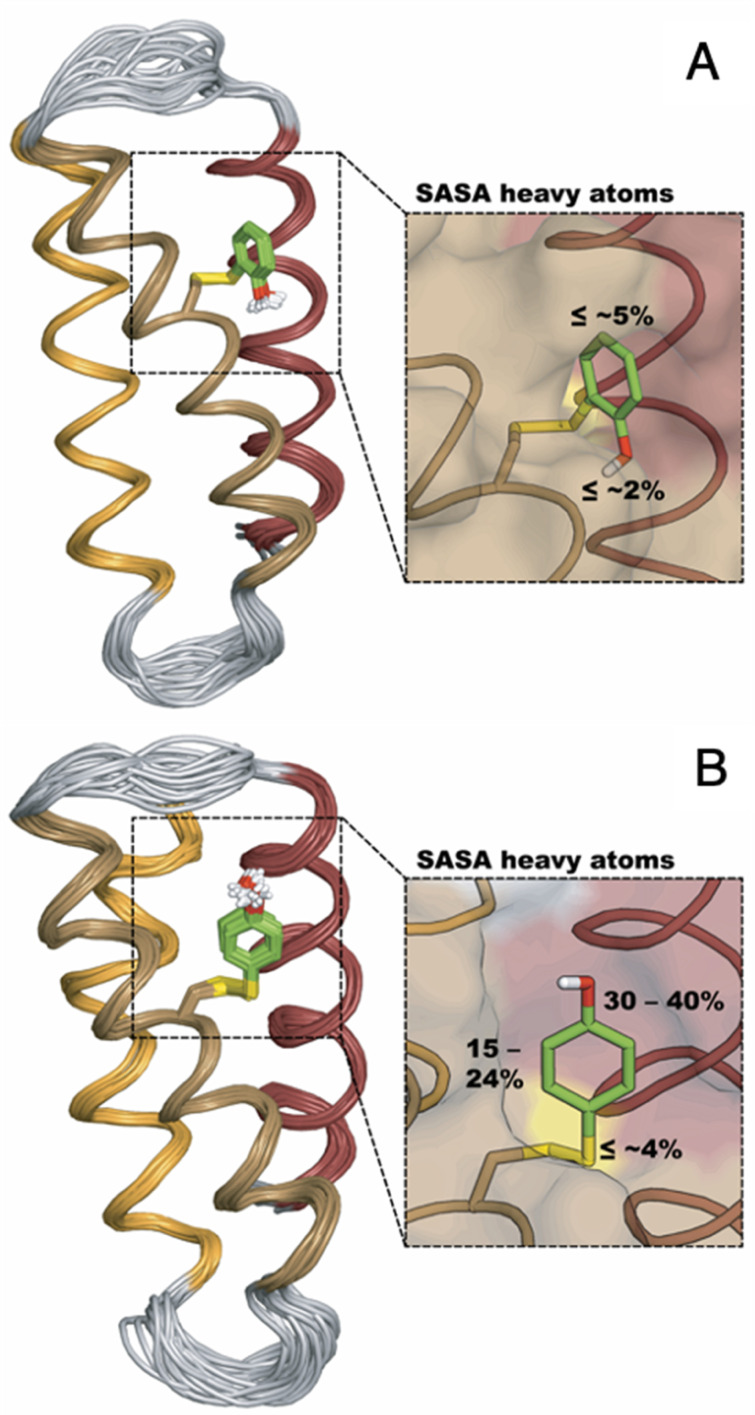
Ribbon diagram representations of the (A) 2MP-α_3_C (RCSB PDB ID 2LXY) and (B) 4MP-α_3_C (8VSW) solution NMR structures. The average SASA of 2MP-C_32_ and 4MP-C_32_ are 3.5 ± 0.7% and 8.7 ± 2.4%, respectively, across the 32-member structural ensembles that represent these proteins in solution. The zoom-in panels display the ensemble average SASA of the heavy atoms in the MP-C_32_ residues. Top panel, SASA of 2MP-C_32_: aromatic C_1_ carbon and phenol oxygen, ≤∼2%; all other heavy atoms, ≤∼5%. Bottom panel, SASA of 4MP-C_32_: aromatic C_1_ carbon and phenol oxygen, 30–40%; all remaining aromatic carbons, 15–24%; S–S bridge and C_32_ atoms, ≤∼4%. The percent SASA given for the heavy atoms are relative to the total area of each individual atom.

The α_3_X system was designed to sequester X_32_ and thereby isolate and stabilize the X_32_˙ state. NMR spectroscopy has been used to obtain high-quality solution structures of α_3_X proteins, including α_3_Y (RCSB PDB ID 2MI7), 2MP-α_3_C (2LXY) and 4MP-α_3_C (this study, see ESI† page S9 for experimental restraints and structural statistics). Y_32_ displays effectively no SASA (0.2 ± 0.2%) and resides at an average depth of 7.7 ± 0.3 Å below the protein surface.^12^ The MP-α_3_C proteins were designed to modulate the exposure of the phenol OH while minimizing other structural changes, both globally to the α_3_ scaffold and locally to the radical site.^9^ As illustrated in Fig. 2, the MP-α_3_C structures confirm this very detailed design and show that the phenol OH SASAs for 2MP-α_3_C and 4MP-α_3_C differ by around one order of magnitude.

**Fig. 3:**
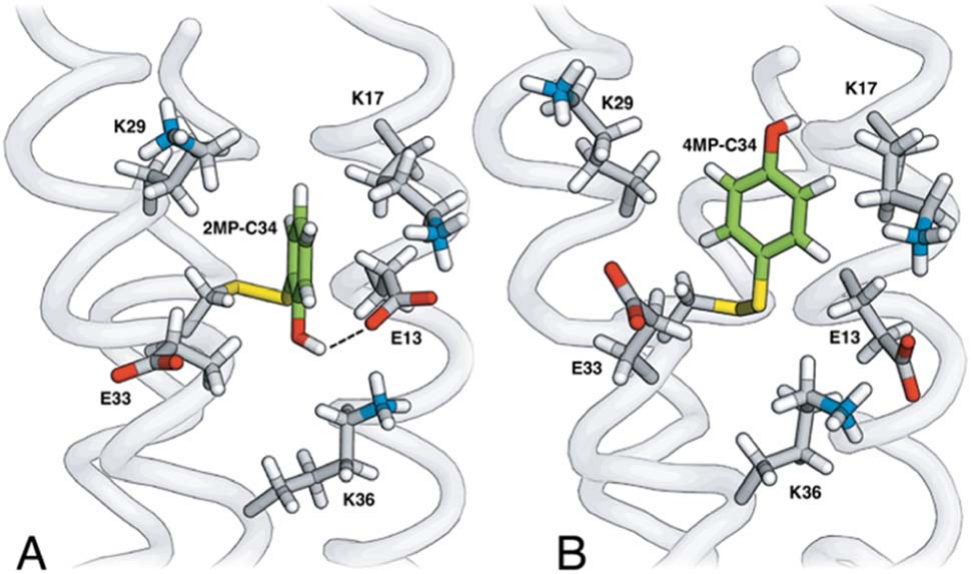
Site 32 in (A) 2MP-α_3_C and (B) 4MP-α_3_C consist of atoms from hydrophobic amino acids (not shown) and five hydrophilic residues, K_17_, K_29_, K_36_, E_13_ and E_33_. The phenol OH points toward (2MP-C_32_) or away from (4MP-C_32_) the carboxylate groups of E_13_ and E_33_. Analysis of the 2MP-α_3_C structure provides a phenol O to glutamate O distance of 3.2 ± 0.5 Å and 7.2 ± 0.2 Å for the 2MP-C_32_/E_13_ and 2MP-C_32_/E_33_ pair, respectively.

Fig. 3 shows the five hydrophilic residues that form part of the MP-C_32_ radical sites. We observe no obvious protein residue that may serve as the primary proton acceptor upon 4MP-C_32_ oxidation. Water and/or buffer species appear more likely. In contrast, the 2MP-C_32_ phenol O and the E_13_ carboxylate group reside at an average distance consistent with a weak H-bond (O–O distance = 3.2 ± 0.5 Å). We hypothesized that the presence of a H-bond could facilitate PT to E_13_ upon 2MP-C_32_ oxidation. To investigate this further, PCET rate constants were determined for 4MP-α_3_C and 2MP-α_3_C ± E_13_ (*vide infra*).

Table 1 summarizes relevant thermodynamic properties of the MP-α_3_C proteins relative to the α_3_Y reference with protein. *E*°′(X_32_˙/X_32_) of 2MP-α_3_C and 4MP-α_3_C are 54 ± 3 and 175 ± 10 mV less oxidizing relative to α_3_Y between pH 5.0 and 10.^5^ The MP-C_32_ residues exhibit p*K*_a_ values that are 1.6–2.1 units below the p*K*_a_ of Y_32_. 2MP-α_3_C has a higher p*K*_a_ value than the other MP-α_3_C proteins, likely because of stabilization from H-bonding to E_13_. In the absence of this interaction, the p*K*_a_ of the phenol OH decreases by 0.5 units.

**Table 1:** α_3_X midpoint reduction potentials, *E*°′, and p*K*_a_ values ± SD^a^

Protein	*E*°′(X_32_˙/X_32_)/mV		*E*°(X_32_˙/X_32_^−^) mV^b^	p*K*_a_
	At pH 5.5	At pH 8.5		
α_3_Y	1065 (±2)	904 (±3)	749 (±4)	11.3 (±0.1)
2MP-α_3_C	1011 (±3)	847 (±2)	780 (±4)	9.7 (±0.2)^c^
4MP-α_3_C	890 (±10)	715 (±10)	654 (±10)	9.5 (±0.1)
2MP-α_3_C-E_13_A	—	—		9.2 (±0.2)^c^

a Potentials (*vs.* NHE) and p*K*_a_ values were obtained from ref. 5.

b Determined from α_3_X Pourbaix diagrams^5^ at pH >> p*K*_a_ of reduced X_32_.

c Determined in the present work (Fig. S1 and S2).

### Radical formation and decay in MP-α_3_C

**Scheme 1:**
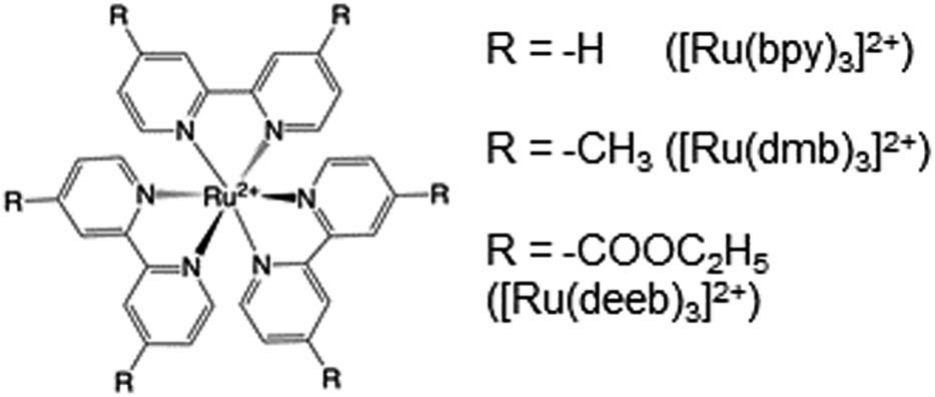
Chemical structure of employed ruthenium tris-4,4′-R-2,2′-bipyridine photosensitizers.

**Fig. 4:**
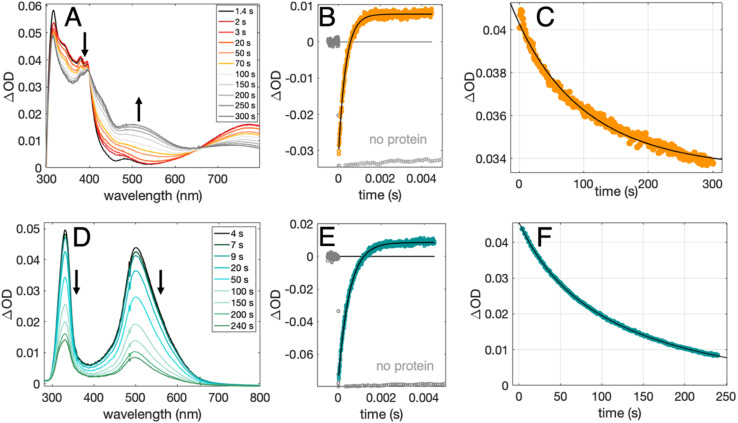
TA spectra, formation kinetics traces, and decay kinetics traces for 2MP˙-C_32_ (top row) and 4MP˙-C_32_ (bottom row). (A and D): TA difference spectra recorded at pH 6.5 (±0.1) following a 500 ms 447.5 nm LED pulse. (B and E): TA kinetic traces recorded at pH 8.5 (±0.1) following a 10 ns laser pulse at 460 nm, monitored at 410 nm (2MP-α_3_C, orange) or 450 nm (4MP-α_3_C, green), and single-exponential fits (black) following the oxidation of MP-C_32_ by [Ru(bpy)_3_]^3+^; traces without protein are shown in gray. (C and F): decay kinetics recorded at pH 6.5 (±0.1) following a 500 ms 447.5 nm LED pulse, monitored at 380 nm (2MP˙-α_3_C), and 500 nm (4MP˙-α_3_C), where black lines show second-order fits. Samples contained 230-590 μM protein, 30 μM [Ru(bpy)_3_]^2+^, and 4–5 mM [Co(NH_3_)_5_Cl]^2+^.

Radical formation and decay were followed by TA spectroscopy. The Ru^3+^ oxidant was formed *in situ via* the flash-quench method^12,13,17,35^ on samples containing MP-α_3_C protein, photosensitizer ([Ru(L)_3_]^2+^, L = 4,4′-R_2_-2,2′-bipyridine, R = –H [Ru(bpy)_3_]^2+^, –CH_3_ [Ru(dmb)_3_]^2+^, or –COOC_2_H_5_ [Ru(deeb)_3_]^2+^, Scheme 1), and quencher ([Co(NH_3_)_5_Cl]^2+^ or persulfate (Na_2_S_2_O_8_)). The photosensitizers used here span a Δ*E*° range of *ca.* 440 mV: *E*°([Ru(dmb)_3_]^3+/2+^) = 1100 mV, *E*°([Ru(bpy)_3_]^3+/2+^) = 1260 mV, and *E*°([Ru(deeb)_3_]^3+/2+^) = 1540 mV; all values reported *vs.* the NHE, see ESI† page S7 for details).^15^ The estimated error for each absolute *E*°(Ru^3+/2+^) value is ∼±30 mV. For radical formation kinetics, a 10 ns laser flash at 460 nm was used to excite the photosensitizer, which in turn was oxidatively quenched to form [Ru(L)_3_]^3+^. The PCET reaction leading to radical formation was followed at 410 and 450 nm, monitoring [Ru(L)_3_]^2+^ ground state bleach recovery concomitant with the growth of radical absorption (Fig. 4B and E). The 410 and 450 nm traces were well fitted with single-exponential functions, following a pseudo-first order dependence on the concentration of [Ru(L)_3_]^3+^, with MP-α_3_C in excess (*vide infra*). Note that without protein, the [Ru(L)_3_]^2+^ ground state bleach was stable on the time scale examined (grey data in Fig. 4B and E). For radical spectra and decay kinetics, a 500 ms LED pulse was instead used to excite the photosensitizer, which was quenched by [Co(NH_3_)_5_Cl]^2+^.

Fig. 4 shows TA spectra and radical formation and decay kinetics for 2MP-α_3_C (top row) and 4MP-α_3_C (bottom row). We note that previous protein film voltammetry (PFV) and TA studies have shown that the α_3_ scaffold is unreactive, even at highly oxidizing conditions.^7,8,12–14,17^ PFV characterization of α_3_X proteins containing Y or Y analogs show fully reversible X_32_ ↔ X_32_˙ + H^+^(bulk) redox cycles.^7,8,10,11,14^ This is due to the large redox-induced p*K*_a_ shifts of phenols, with the p*K*_a_ of the cation radical typically <0.^36^ The TA spectra shown in Fig. 4A and D are thus assigned to the neutral 2MP˙-C_32_ and 4MP˙-C_32_ radicals, respectively. Consistent with this conclusion, the 2MP˙-C_32_ spectra are reminiscent of Y_32_˙ spectra recorded under similar conditions,^12^ while the 4MP˙-C_32_ spectra share spectral similarities with the neutral 4-hydroxythiophenoxyl radical.^37^

Radical decay kinetics were extracted by plotting the change in radical absorption (380 nm for 2MP-α_3_C and 500 nm for 4MP-α_3_C) as a function of time. A fit to second order kinetics was used to calculate the first half-life, *t*_1/2_, where *t*_1/2_ = 1/(k_2_Abs_0_(MP˙-C_32_)). This analysis provided *t*_1/2_(2MP˙-C_32_) = 100 s, *t*_1/2_(4MP˙-C_32_) = 130 s, and *t*_1/2_(2MP˙-C_32_ in 2MP-α_3_C-E_13_A) = 24 s (Fig. S10†). Extinction coefficients are not known for these radicals, but we estimated the initial concentrations, and thus calculated the rate constants. From the initial Ru^2+^ ground state bleach and final radical signal in Fig. 4B and E, and assuming ∼100% conversion to the radicals, the initial radical concentration is ∼13 μM in the experiments with pulsed diode excitation (Fig. 4A, C, D and F; see ESI† for details). This estimate gives rate constants for radical–radical decay of *k*_2_ ≈ 80, 60 and 300 M^−1^ s^−1^ for 2MP˙-C_32_, 4MP˙-C_32_, and 2MP˙-C_32_ in 2MP-α_3_C-E_13_A, respectively. Both 2MP˙-C_32_ and 4MP˙-C_32_ give rise to optical features that persisted for more than 200 s. This provided the opportunity to collect EPR spectra, as describe below.

### Characterization of the 2MP-α_3_C and 4MP-α_3_C radicals

**Fig. 5:**
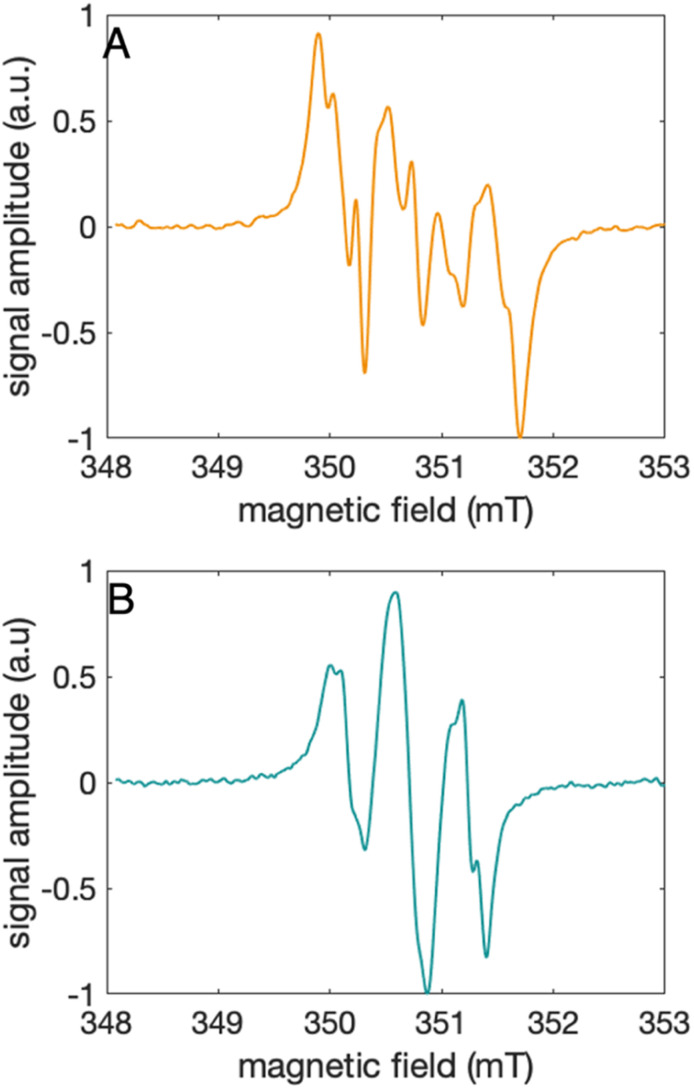
EPR spectra collected at ambient temperature under continuous illumination using a 447.5 nm LED of (A) 2MP-α_3_C, shown in orange, and (B) 4MP-α_3_C, shown in green. EPR settings: microwave frequency, 9.85 GHz; microwave power 6.3 mW; modulation frequency 100 kHz; modulation amplitude 0.1 mT.

The distinct UV-vis spectra shown in Fig. 4A and D suggest that 2MP˙-α_3_C and 4MP˙-α_3_C have significantly different electronic structures. To support the notion that each UV-vis spectrum represents a single major radical species, we used EPR spectroscopy to further characterize MP-α_3_C under photo-oxidizing conditions. EPR spectra were collected from MP-α_3_C dissolved in 100 mM KP_i_, 40 mM KCl pH 6.5 buffer, and using [Ru(bpy)_3_]^2+^ as the photosensitizer and [Co(NH_3_)_5_Cl]^2+^ as the quencher. The experiments were conducted at room temperature under constant illumination by a 447.4 nm LED lamp. A strong paramagnetic signal was observed to rise when the LED lamp was switched on and to subsequently decay when the LED lamp was switched off. No signal was observed prior to illumination. The EPR spectra representing the light-induced 2MP˙ and 4MP˙ species are shown in Fig. 5A and B, respectively. The spectra are consistent with the primary radicals 2MP˙ and 4MP˙. The former give rise to a hyperfine pattern due to hydrogen nuclear spin of four inequivalent protons, while the latter contains two equivalent proton pairs. As expected, the widths of the MP-α_3_C spectra are narrow relative to a typical protein Y˙ spectrum. The MP-C_32_ residues lack β-methylene protons (Fig. 1), which have a major geometry-dependent impact on the linewidth of a Y˙ spectrum.^38^

**Fig. 6:**
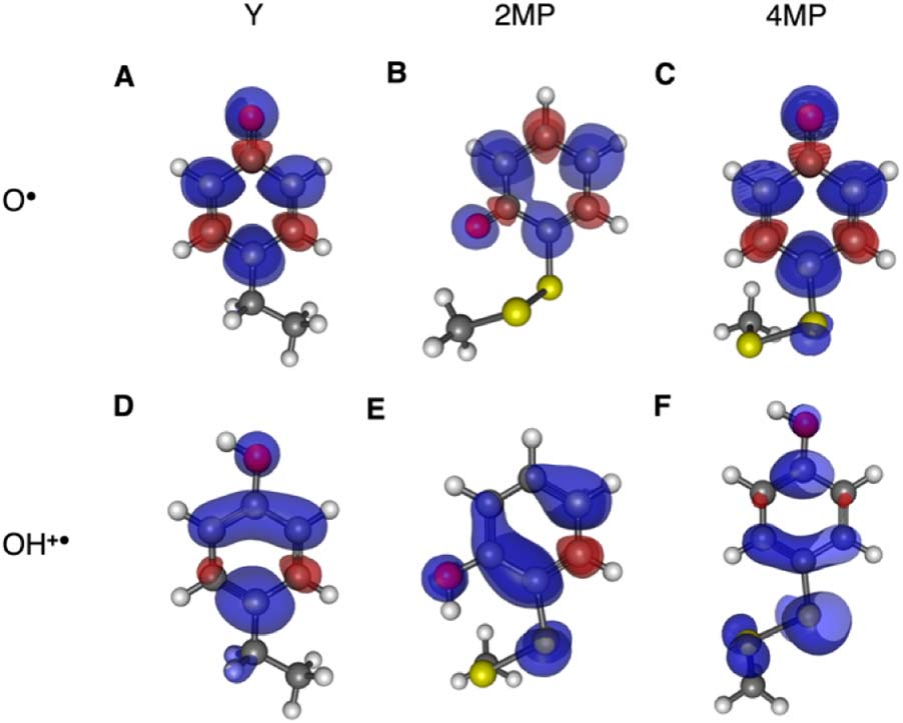
Spin densities computed with CASSCF/aug-cc-pVTZ for neutral radicals (A–C) and cationic radicals (D–F) with an isovalue of 0.002 Å^−3^. Analogous plots for DFT are provided in the ESI.†

The radical spin distribution was investigated using both DFT and multireference methods. The spin densities were calculated for optimized geometries of the 2MP-α_3_C, 4MP-α_3_C, and α_3_Y side chain analogs in their neutral and cationic radical states using unrestricted DFT and CASSCF calculations. Spin densities were visualized, and the Mulliken spin population^39^ values were computed. These are provided for the CASSCF/aug-cc-pVTZ computations in Fig. 6 and Table 2. Fig. 6 shows the expected alternating pattern of α and β spin density in the aromatic ring for neutral radicals, with cationic radicals having less spin density on the oxygen and being more delocalized over the aromatic ring. This behavior is quantified in Table 2 through Mulliken spin population analysis. These trends show that the neutral radical 4MP˙ has slightly more spin on the sulfur atoms than 2MP˙, but this effect is much more pronounced in the radical cation forms. Values for the spin populations computed for the Y neutral and cationic radicals are also provided as a reference. These trends were also observed for DFT calculations using three different functionals, namely B3LYP-D3(BJ),^23,24,40^ ωB97X-D,^25^ and M06-2X,^26^ with the 6-31G** and 6-31+G** basis sets for all three functionals and additionally the 6-31++G** basis set^41–43^ for the ωB97X-D functional (see ESI†). We conclude that for the neutral radicals there is very little spin density on the sulfur atoms that ligate the phenols to the α_3_ scaffold (we note that early calculations suggested a larger spin density on the sulphur).^37^ Our results strengthen the use of MPs as a model system for canonical protein Y redox sites.

**Table 2:** Mulliken spin populations on key atoms of side chain analogs of the redox-active side chain in 2MP-α_3_C and 4MP-α_3_C calculated with CASSCF/aug-cc-pVTPZ

System	Atom^a^			Total S^b^
	O	S	S_2_	
Y–O˙	0.340	—	—	—
2MP-O˙	0.310	0.013	0.006	0.019
4MP-O˙	0.321	0.049	0.002	0.051
Y–OH^+^˙	0.106	—	—	—
2MP-OH^+^˙	0.120	0.105	0.003	0.108
4MP-OH^+^˙	0.041	0.453	0.078	0.531

a O refers to the hydroxyl oxygen of the sidechain, S refers to the sulfur atom closest to the phenol ring, and S_2_ refers to the sulfur atom most distal to the phenol ring, *i.e.*, closest to the backbone.

b “Total S” refers to the total spin population on the sulfur atoms in the molecule. A full set of data is available in the ESI for all theoretical methods used in this study.

### pH-dependent rate constants for radical formation

**Fig. 7:**
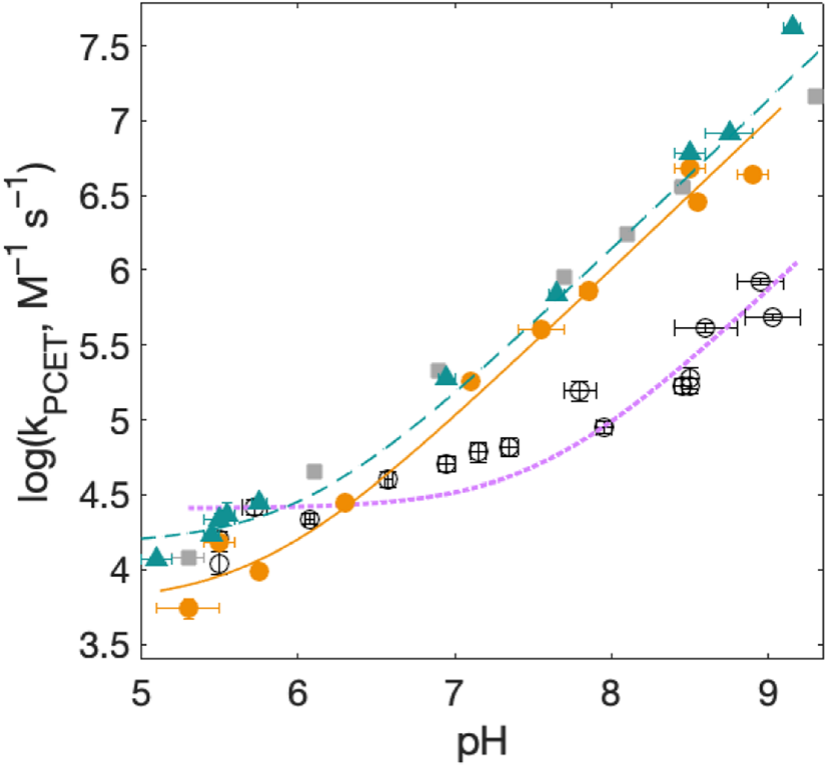
Rate constants for radical formation *vs.* pH using [Ru(bpy)_3_]^3+^ as oxidant for 2MP-α_3_C (orange dots), 2MP-α_3_C-E_13_A (grey squares), and 4MP-α_3_C (green triangles), compared to previously published data for α_3_Y (black circles).^13^ Standard deviations are shown, but are often smaller than the size of the data symbols. Samples contained 60–620 μM protein, 30 μM [Ru(bpy)_3_]^2+^, and 4–6 mM [Co(NH_3_)_5_Cl]^2+^. Fits according to eqn (1) are shown as lines (see Table 4 for results).

**Fig. 8:**
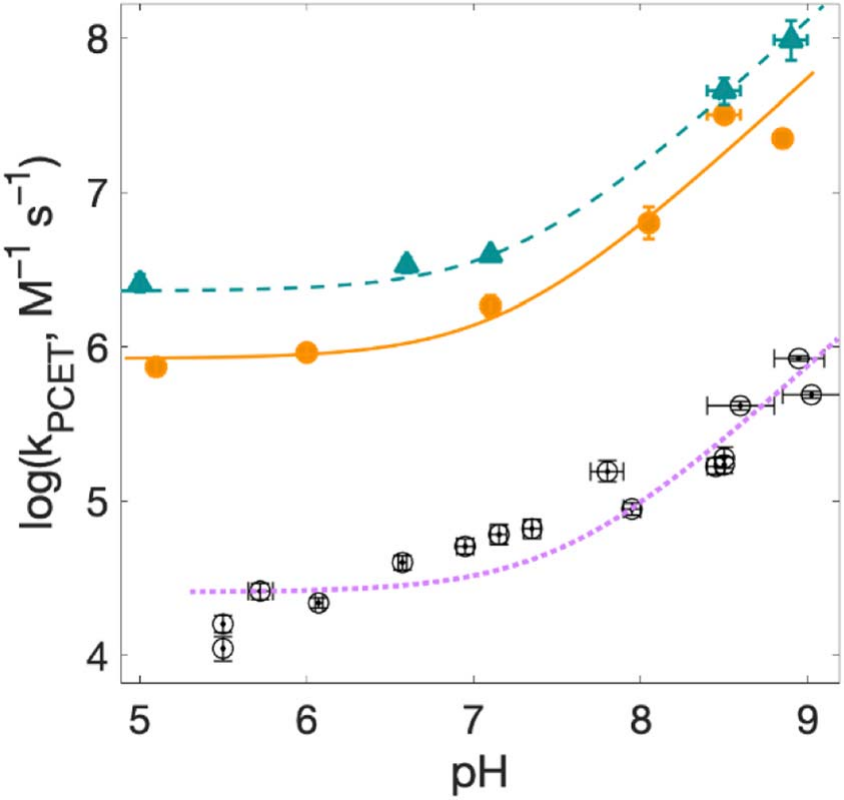
Rate constants for radical formation as a function of pH for 2MP-α_3_C (orange dots) and 4MP-α_3_C (green triangles), with [Ru(deeb)_3_]^3+^ as oxidant, and α_3_Y (black circles, fit with a purple dotted line curve) with [Ru(bpy)_3_]^3+^ as oxidant from ref. 13, other conditions as in Fig. 7. Standard deviations are shown, but are often smaller than the size of the data symbols. Fits according to eqn (1) are shown (see Table 4 for results).

PCET rate constants (*k*_PCET_) reflecting MP-C_32_ oxidation by Ru(L)_3_^3+^ were obtained as a function of buffer concentration (Fig. S5†), pH, and 
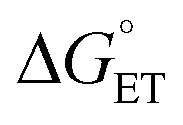
 (Fig. 7 and 8; Table 3). 
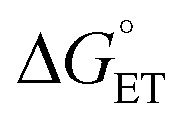
 was modulated by using the three photosensitizers shown in Scheme 1 (Δ*E*°(Ru^(3+/2+)^) *ca.* 440 mV, *vide supra*). In all experiments, the protein concentration was much higher (200–600 μM) than the concentration of the *in situ* generated Ru^3+^ oxidant (1–6 μM), resulting in pseudo-first order kinetics for radical formation. To confirm that the reactions were first order with regards to [protein], rate constants were also determined as a function of [protein] at one or two pH values for each oxidant (Fig. S4, S7, and S9; see ESI† for details). The large excess of protein also resulted in complete consumption of [Ru(L)_3_]^3+^, with no significant remaining Ru^2+^ bleach. Thus, the reverse reaction could be ignored even for the reactions where Δ*G*° ≈ 0 and the observed rate constant can be identified as the forward rate constant for PCET, *k*_PCET_ (see General discussion).

**Table 3:** PCET rate constants at pH 5.5 and 8.5 for 2MP-α_3_C and 4MP-α_3_C with [Ru(L)_3_]^3+^ oxidants and their Ru^3+/2+^ potentials

Oxidant	pH	*k*_PCET_ (M^−1^ s^−1^)	*E*°^a^ (mV *vs.* NHE)
**2MP-α**_**3**_ **C**			
Ru(dmb)_3_^3+^	5.5(±0.1)	2.8 × 10^3^	+1100
Ru(bpy)_3_^3+^	5.5(±0.1)	1.5 × 10^4^	+1260
Ru(dmb)_3_^3+^	8.5(±0.1)	7.7 × 10^5^	+1100
Ru(bpy)_3_^3+^	8.5(±0.1)	4.8 × 10^6^	+1260
Ru(deeb)_3_^3+^	8.5(±0.1)	3.2 × 10^7^	+1540

**4MP-α**_**3**_ **C**			
Ru(dmb)_3_^3+^	5.5(±0.1)	4.4 × 10^3^	+1100
Ru(bpy)_3_^3+^	5.5(±0.1)	2.1 × 10^4^	+1260
Ru(dmb)_3_^3+^	8.5(±0.1)	1.5 × 10^6^	+1100
Ru(bpy)_3_^3+^	8.5(±0.1)	6.1 × 10^6^	+1260
Ru(deeb)_3_^3+^	8.5(±0.1)	5.4 × 10^7^	+1540

a Error bars are estimated to be ±30 mV.

There is no significant change in *k*_PCET_ derived from 2MP-α_3_C and 4MP-α_3_C samples containing 20 to 400 mM KP_i_ (Fig. S5†). This observation shows that a buffer species does not serve as the primary acceptor of the phenolic proton as 2MP-C_32_ or 4MP-C_32_ is oxidized. These results agree with results for α_3_Y, where buffer species were shown to not participate in the PCET reaction,^13^ but stand in stark contrast to results obtained for small molecule Y and W derivatives in solution, where common buffers may be the primary acceptor even at moderate concentrations (≳10 mM).^44,45^

Fig. 7 shows *k*_PCET_ as a function of pH using [Ru(bpy)_3_]^3+^ as the oxidant for 2MP-α_3_C (orange), 2MP-α_3_C-E_13_A (grey), and 4MP-α_3_C (green) compared to previously published data on α_3_Y (black/purple). Kinetic data for all proteins are fit with eqn (1) (below), and the resulting *k*_PCET_ values are given in Table 4.

1 *k*_PCET_ = *k*_YOH_ + *k*_YO^−^_ × 10^(pH-p*K*_a_)^

**Table 4:** Rate constants *k*_YOH_ and *k*_YO^−^_a

Oxidant	*k*_YOH_ (M^−1^ s^−1^)	*k*_YO^−^_ (M^−1^ s^−1^)
**2MP-α**_**3**_ **C**		
Ru(dmb)_3_^3+^	—	1.2 × 10^7^^b^
Ru(bpy)_3_^3+^	5.8 × 10^3^	5.1 × 10^7^
Ru(deeb)_3_^3+^	8.4 × 10^5^	2.7 × 10^8^

**4MP-α**_**3**_ **C**		
Ru(dmb)_3_^3+^	—	1.5 × 10^7^^b^
Ru(bpy)_3_^3+^	1.5 × 10^4^	4.4 × 10^7^
Ru(deeb)_3_^3+^	2.3 × 10^6^	4.0 × 10^8^

**α**_**3**_ **Y**		
Ru(bpy)_3_^3+^	2.6 × 10^4^	1.4 × 10^8^

a From fits according to eqn (1).

b Calculated from the pH 8.5 value multiplied by 10^(p*K*_a_−8.5)^, see text.

For α_3_Y, the first, pH-independent term that dominates at low pH is assigned to a concerted CEPT reaction with water as the primary proton acceptor.^13^ The second term is assigned to pre-equilibrium PTET (PTET_pre-eq_) with the equilibrium fraction of the Y–O^−^ species increasing ten-fold per pH unit. For MP-α_3_C, the contribution of the first term is very small and is only noticeable as a weak pH dependence for the lowest pH data points. As shown in the next section, the mechanisms can be assigned in complete analogy to the α_3_Y system: CEPT with H_2_O as proton acceptor at the lowest pH values, and PTET at the higher pH values.

### Assigning the PCET mechanisms

The kinetic isotope effect (KIE) on PCET rates was determined at 2–3 different pL (L = H or D) values using [Ru(bpy)_3_]^3+^ as oxidant. The observed KIE values were significant: 2.9 (pL 6.0 ± 0.1) and 3.6 (pL 9.0 ± 0.1) for 2MP-α_3_C, and 6.2 (pL 5.3 ± 0.3), 11.2 (pL 6.3 ± 0.1), and 13.5 (pL 8.6 ± 0.1) for 4MP-α_3_C. The large KIE values confirm that PT is part of the rate-limiting step. From these KIEs, we can exclude an ET-limited ETPT reaction over the entire pH range examined. We can also exclude a pre-equilibrium ETPT because this mechanism requires that the pre-equilibrium is faster than the subsequent reaction, which is highly improbable given that the p*K*_a_'s of phenols typically drop to values <0 upon oxidation.^36^

A pH-independent PTET reaction at low pH can be excluded because deprotonation of weak acids to water (H_2_O) is slow: *k*_PT_ ∼100 s^−1^ for p*K*_a_ = 9,^46^ which is much slower than our observed first-order rate constants. Other potential proton acceptors (OH^−^, buffer) increase in concentration as the pH increases and would not have given a pH-independent rate constant. This analysis suggests that the pH-independent reaction is CEPT with H_2_O as the primary proton acceptor.

The pH-dependent rate constants (second term in eqn (1)) can have two origins. First, for a PTET_pre-eq_ mechanism, the pre-equilibrium shifts with pH because at higher pH values there is a larger fraction of already deprotonated species, which leads to faster rate constants. Second, for an irreversible reaction step (CEPT or PT-limited PTET), the concentration of the proton-accepting species can depend on pH, which would be the case for *e.g.* OH^−^ and base forms of the buffer. At high pH, CEPT with OH^−^ as the primary proton acceptor can most likely be excluded because the observed (pseudo-first order) rate constants are too large to be explained by a diffusional reaction with the [OH^−^] present in the solution in the pH interval studied, see ESI page S12.† The PT-limited PTET could be excluded by comparing the rate constants with those obtained with a weaker oxidant, namely [Ru(dmb)_3_]^3+^ at pH 5.5 ± 0.1 and 8.5 ± 0.1 (Table 3). Both 2MP-α_3_C and 4MP-α_3_C showed slower rate constants with the weaker oxidant. This is inconsistent with a PT-limited reaction but is consistent with PTET_pre-eq_, for which the overall rate constant depends also on the rate constant for the second step. This analysis suggests that 2MP-α_3_C and 4MP-α_3_C oxidation by [Ru(bpy)_3_]^3+^ (Fig. 7) proceeds mainly *via* a PTET_pre-eq_ mechanism, with CEPT dominating only at the lowest pH values. Note that we can exclude a significant contribution from the reverse PCET reaction for reactions at Δ*G*° ≈ 0, which could have given a pH-dependence of the net reaction,^47^ because we use great excess of protein, making the reaction go to completion in a single kinetic phase (Δ*G* < 0), and no remaining Ru^2+^ bleach is seen over a large variation of pH values and observed rate constants. Moreover, a parallel pH-dependence is observed also with the strong oxidant [Ru(deeb)_3_]^3+^, for which Δ*G*° ≪ 0.

### Proton transfer is not facilitated by increased solvent exposure nor by a nearby internal proton acceptor

Rate constants determined for 4MP-α_3_C are slightly higher compared to those for 2MP-α_3_C. This is most likely due to the lower *E*°′(X_32_˙/X_32_) and p*K*_a_ values of 4MP-α_3_C. As the rate constant difference is modest, it appears that the higher phenol OH SASA of 4MP-C_32_ does not further accelerate PCET. Specifically, it does not seem to facilitate PT to water, and it does not allow sufficient access of buffer for this to be the primary proton acceptor.

The relatively close distance of 2MP and E_13_ in 2MP-α_3_C (Fig. 3A) did not lead to any clear increase of the PCET rate constant compared to 4MP-α_3_C. Glutamate is a stronger base than water, with p*K*_a_ ∼4.5 *vs.* 0 for their respective conjugate acids, and glutamate as a proton acceptor would be expected to accelerate PCET. Moreover, the 2MP-α_3_C rate constant is even slightly higher for 2MP-α_3_C-E_13_A, which lacks this glutamate residue (Fig. 7). We can therefore exclude E_13_ as the primary proton acceptor and instead assign water as the likely proton acceptor for 2MP-α_3_C, just as for the other proteins.

MD simulations were performed on the MP-α_3_C solution NMR structures (Fig. 2) to better understand the H-bonding interaction between the 2MP-C_32_ or 4MP-C_32_ and water, E_13_, or E_33_ (Fig. 3 and Table S12†). The simulations show that 4MP-C_32_ H-bonds primarily with water and has negligible interaction with E_13_ and E_33_. 2MP-C_32_ H-bonds with water to a much lesser extent and interacts also with E_33_ but not significantly with E_13_. Note that the NMR structure does not indicate a H-bond between 2MP-C_32_ and E_33_ (Fig. S21†), within strict distance and angle criteria. Moreover, the H-bond between 2MP-C_32_ and E_13_ is retained in a QM/MM MD trajectory, where the 2MP-C_32_ and E_13_ sidechains are treated with DFT (Fig. S23†). Thus, the preference for H-bonding of 2MP-C_32_ to E_33_ over E_13_ may be due to limitations of the force field, and the QM/MM simulations suggest that 2MP-C_32_ can H-bond to E_13_. Nevertheless, analysis of the rate constants suggests that although E_13_ is within H-bonding distance to 2MP-C_32_, other factors such as insufficient proton vibrational wavefunction overlap inhibit PT.

### Rate constants for PCET with various oxidation strengths

For solvated small-molecule Y compounds, a stronger oxidant has been shown to change the PCET mechanism from PTET to CEPT.^2^ To test whether an external oxidant could change the PCET mechanism in the α_3_X protein system, rate constants were determined as a function of pH using the stronger oxidant [Ru(deeb)_3_]^3+^ with persulfate as the quencher (Fig. 8; see ESI† for details). This gave much faster PCET rate constants, and a much more prominent contribution from the pH-independent CEPT reaction (first term in eqn (1)). The pH dependence is very similar to what was observed for α_3_Y using [Ru(bpy)_3_]^3+^ as the oxidant (black data with purple dotted fit in Fig. 8).^13^ Fitting the data to eqn (1) yielded rate constants for the protonated and deprotonated fractions of MP-C_32_ (Table 4).

With [Ru(deeb)_3_]^3+^ as the oxidant, the rate constants at pH >7 increase with pH, analogous to the data with [Ru(bpy)_3_]^3+^. The mechanism can thus be assigned to PTET_pre-eq_ also with the stronger oxidant; PT-limited PTET and CEPT with OH^−^ as the proton acceptor can be excluded as for the experiments with [Ru(bpy)_3_]^3+^ above. The rate constant *k*_YO^−^_ is higher with [Ru(deeb)_3_]^3+^, as expected from the driving force dependence of Y–O^−^ oxidation (see General discussion below).

Fig. 8 shows PCET rate constants of 2MP-α_3_C and 4MP-α_3_C with the stronger oxidant [Ru(deeb)_3_]^3+^, in comparison to α_3_Y using [Ru(bpy)_3_]^3+^. The general trend in pH-dependence of PCET rate constants for 2MP-α_3_C and 4MP-α_3_C *versus* α_3_Y is similar, but the rate constants are significantly accelerated in the former. At low pH, the use of a stronger oxidant can favor the ETPT mechanism, and it is important to demonstrate that proton transfer is a part of the rate limiting step. Experiments were therefore repeated in D_2_O at pL 5.6(±0.1) with [Ru(deeb)_3_]^3+^ as the oxidant, and resulted in KIE ∼3 for both 2MP-α_3_C and 4MP-α_3_C. This large KIE excludes an ET-limited ETPT mechanism and suggests that PCET proceeds *via* CEPT with H_2_O as the primary proton acceptor, as was the case with the weaker oxidants. The rate constant is much larger, as can be expected with the much stronger oxidant (see next section). We note that the pH-independent rate constant cannot be explained by formation of an internal H-bond for 2MP-C_32_ since 4MP-C_32_ shows the same behavior without having a nearby protein proton acceptor.

## General discussion

### Changing the PCET mechanism by tuning the driving force

**Fig. 9:**
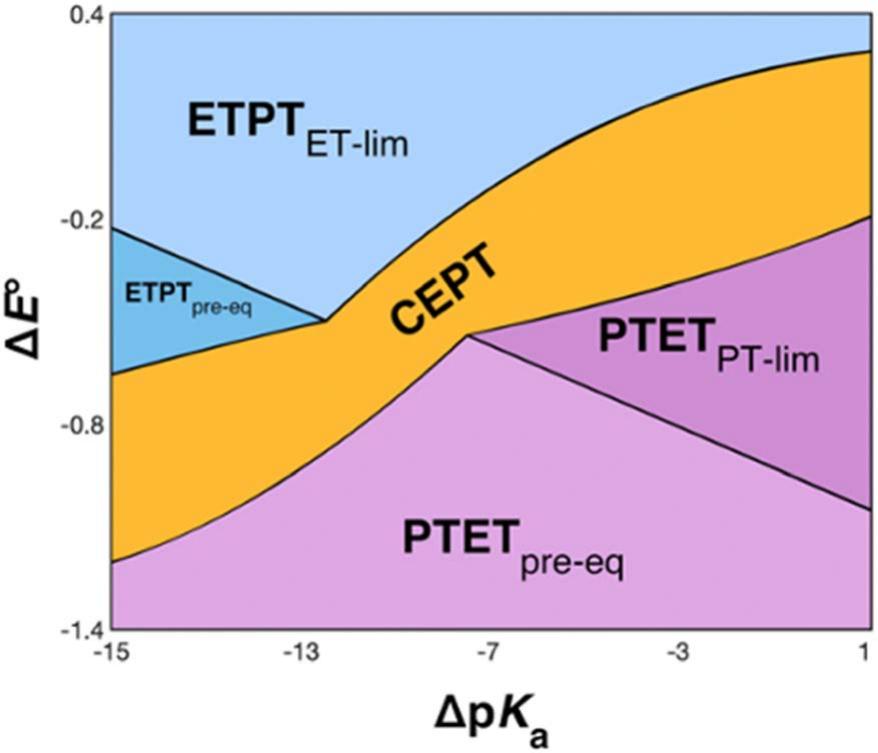
Schematic zone diagram for a PCET oxidation of a compound HA to A˙, where the reaction exhibits a sufficiently large vibronic coupling that CEPT can compete in this range of Δ*E*° and Δp*K*_a_ values. The axes are defined as Δ*E*° = *E*°(oxidant) − *E*°(HA˙^+^/HA) (in units of volts) and Δp*K*_a_ = p*K*_a_(H^+^ base) − p*K*_a_(HA), so that the overall driving force for PCET increases when moving upwards and to the right in the diagram. Adapted with permission from ref. 1. Copyright © 2021 American Chemical Society.

The recently introduced PCET zone diagrams^1^ can help to visualize which mechanism dominates a PCET reaction, depending on the driving force for initial ET or PT (represented by Δ*E*° and Δp*K*_a_, respectively), see Fig. 9. The diagrams assume a Marcus-type free-energy dependence of the rate constant for each mechanistic step (ET, PT or CEPT; see eqn (2) below). The size and shape of each of the mechanistic regions are dictated by the relative pre-exponential factor and reorganization energies for each mechanism.^1^ The schematic zone diagram in Fig. 9 has a large CEPT region, which is a result of two factors. First, a large energetic interdependence of ET and PT, manifested by a large difference in *E*°(X_32_˙^+^/X_32_) *vs. E*°′(X_32_˙/X_32_^−^) and a correspondingly large difference in the phenol p*K*_a_ of oxidized and reduced X_32_, favors a CEPT mechanism under a wide range of conditions. Secondly, a large vibronic coupling between the reactant and product state for CEPT allows for a high probability of electron and proton tunneling. With sufficient kinetic data as a function of Δ*E*° and Δp*K*_a_ for a system, the lines dividing the zones can be quantitatively estimated.^48^

For bimolecular PCET reactions, bases and oxidants of different strengths can be used to access different PCET regions. However, the protein shields the X_32_ pocket and excludes negatively charged buffer species. This is evidenced by our previous studies on α_3_Y^12,13^ as well as the independence of *k*_PCET_ rate constants on the buffer concentration in the present data. Having shown that 2MP-α_3_C is similar to α_3_Y in that X_32_ exhibits a low SASA, comparison between the two proteins allows us to investigate the effect of altering the PT driving force. The p*K*_a_ value is 1.6 lower for 2MP-α_3_C, giving an increase in PT driving force of 95 meV. A change in rate constant by one order of magnitude per p*K*_a_ unit is expected for a PTET_pre-eq_ reaction. Indeed, in the high pH region of Fig. 7, the difference in *k*_PCET_ between 2MP-α_3_C and α_3_Y is between one and one and a half orders of magnitude, as expected.

*E°*(X_32_˙/X_32_^−^) is very similar for 2MP-α_3_C (780 ± 4 mV) and α_3_Y (749 ± 4 mV). Instead, the lower p*K*_a_ value for 2MP-α_3_C changes the PT driving force and would mean moving to the right in Fig. 9 for 2MP-α_3_C compared to α_3_Y. This is consistent with moving from the CEPT region to the PTET_pre-eq_ region. Indeed, this is what is experimentally observed, as CEPT dominates at low pH for α_3_Y, while PTET_pre-eq_ dominates over almost the entire pH range for 2MP-α_3_C with the moderately strong oxidant [Ru(bpy)_3_]^3+^ (Fig. 7). When the ET driving force increases by *ca.* 280 meV by using the much stronger oxidant [Ru(deeb)_3_]^3+^, we move upwards in the diagram and re-enter the CEPT region, as is shown by the data in Fig. 8. 4MP-α_3_C shows an entirely parallel behavior as compared to 2MP-α_3_C; the *ca.* 125 mV lower *E*°(X_32_˙/X_32_^−^) value is not sufficient to favor CEPT with the weaker oxidants, whereas the much greater difference in Δ*E*° with [Ru(deeb)_3_]^3+^ has this effect.

From the more qualitative discussion of changing mechanisms (above), we continue by drawing quantitative comparisons of the rate constants between the different combinations of protein and oxidant with the aid of theories for ET and CEPT. A simplified expression for the free-energy dependence of the rate constant for ET or CEPT is given in eqn (2).^49^2
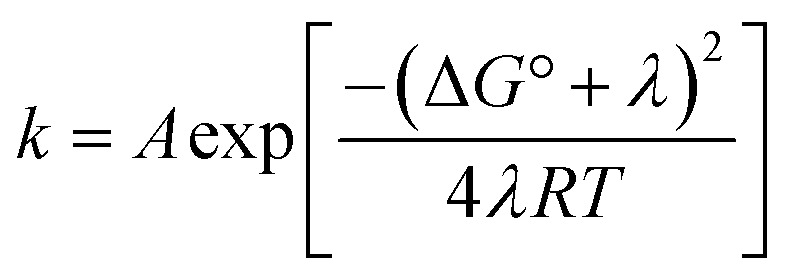


A CEPT reaction may have contributions to the rate constant from transitions to and from several proton vibrational states that can modify the free-energy prediction of eqn (2). For simplicity these effects will be neglected in the present analysis.^50^ The derivative in eqn (3) shows the predicted slope of a typical plot of ln *k vs.* driving force, where ∂ ln(*k*_CEPT_)/∂(−Δ*G*°) = (50 meV)^−1^ when Δ*G*° = 0.3
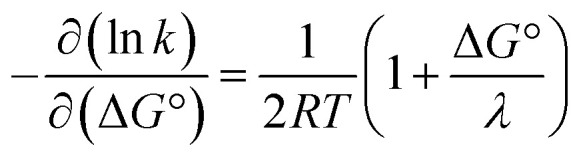


The driving force for CEPT is given by 
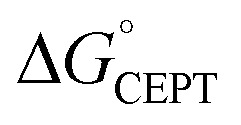
 = −*F*(*E*°(Ru^III^/Ru^II^) *− E*°′(X_32_˙/X_32_)) at pH = p*K*_a_ of the conjugate acid of the proton acceptor; in these systems water is the proton acceptor, and p*K*_a_ (H_3_O^+^) = 0. *E*°′(X_32_˙/X_32_)_pH0_ can be predicted from the Pourbaix diagrams of α_3_Y, 2MP-α_3_C, and 4MP-α_3_C^5,9^ and give 
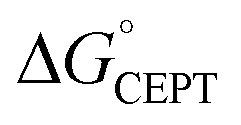
 ≈ 110, 55 and −50 meV, respectively, when [Ru(bpy)_3_]^3+^ is the oxidant. The PCET reaction is observed even when 
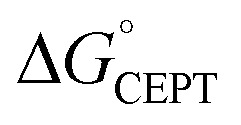
 > 0 because the reaction is driven to completion by the more than 100-fold excess of protein *vs.* [Ru(bpy)_3_]^3+^ generated per laser flash (*ca.* 1–6 μM), Fig. 4B and E.

As described above, the PCET mechanism is consistent with CEPT when *k*_PCET_ is pH-independent (at low pH-values, Fig. 7 and 8). The use of [Ru(deeb)_3_]^3+^*in lieu* of [Ru(bpy)_3_]^3+^ increases 
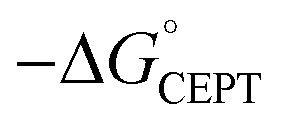
 by 280 meV, and *k*_YOH_ for 2MP-α_3_C and 4MP-α_3_C increase by *ca.* two orders of magnitude (Table 4). This corresponds to a slope according to eqn (3) of (60 meV)^−1^, consistent with CEPT in the normal region with a small driving force.

Shifting the oxidant strength in the CEPT region gives rise to predictable changes in *k*_PCET_ for each protein individually. Comparing the trend in *k*_PCET_ between the different proteins, however, does not consistently correlate with changes in 
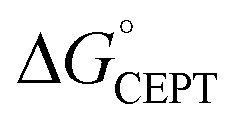
. Notably, α_3_Y has the least favorable 
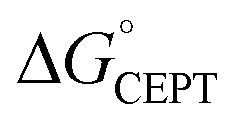
, and yet its CEPT rate constants are the largest (see Fig. 7, low pH region). 4MP-α_3_C, which exhibits the most favorable 
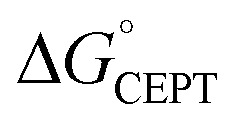
 and which is predicted to have its phenol OH consistently in contact with water (Fig. 3, Table S12†), does not have the largest CEPT rate constants, though they are greater than rate constants for 2MP-α_3_C. From the study of CEPT in this homogeneous series of proteins, it is clear that factors outside of the driving force influence CEPT rate constants. One possibility is that the vibronic coupling varies significantly among the protein systems due to different proton donor–acceptor distances, which influence the overlap between the proton vibrational wavefunctions. As described above, water is assigned as the dominant primary proton acceptor for the α_3_X proteins investigated here. We have previously identified local side chain motions near the Y_32_ site that permit transient access of one to two water molecules to within H-bonding distance of the phenol OH.^13^ 2MP-α_3_C and 4MP-α_3_C behave in a similar manner (Table S12†). The observed differences in the concerted PCET kinetics indicate that α_3_Y can access a state or states where the phenol OH and the water proton acceptor are more optimally oriented with respect to PT relative to the MP-α_3_C proteins.

**Fig. 10:**
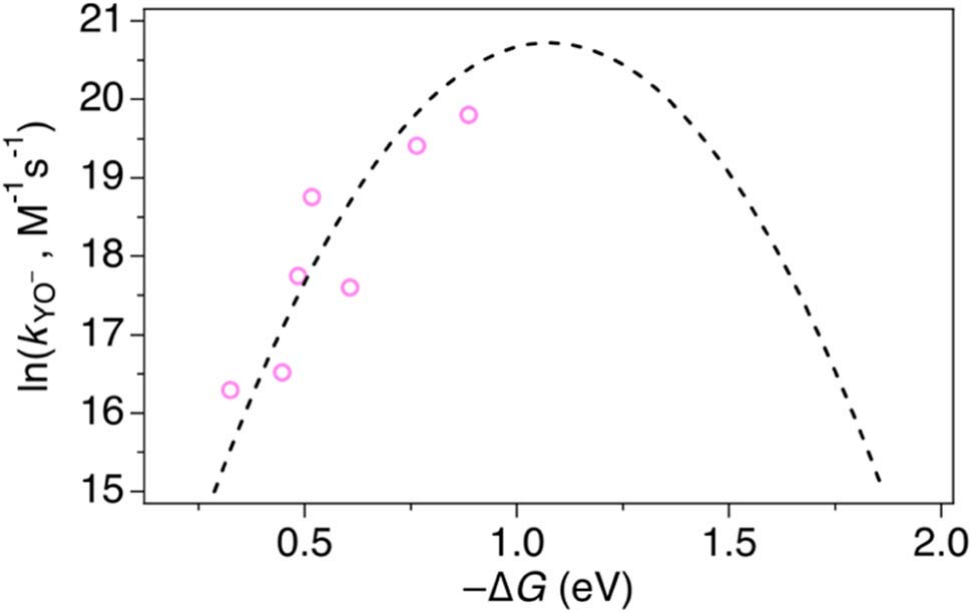
Natural logarithm (ln) of *k*_YO^−^_ rate constants (pink circles) as a function of driving force for ET from YO^−^ to the [Ru(L)_3_]^3+^ oxidant. The dashed line shows a fit according to eqn (2) assuming *A* = 1 × 10^9^ M^−1^ s^−1^, which gave *λ* = 1.1 eV.

At high pH-values, all combinations of protein and oxidant reacted *via* PTET_pre-eq_. The observed rate constants are proportional to the ET rate constant from deprotonated Y_32_ or MP-C_32_ (*k*_YO^−^_ in eq. (1)), and thus depend on the driving force for ET. The values of *k*_YO^−^_ taken from the fits to eqn (1) in Fig. 7 and 8 are plotted *vs.* the driving force for ET from Y–O^−^ to Ru(L)_3_^3+^ (Fig. 10). The *k*_YO^−^_ values for MP-α_3_C/[Ru(dmb)_3_]^3+^ are taken from the rate constants at pH 8.5 and multiplying with 10^(p*K*_a_−8.5)^, *i.e.* assuming that only the second term of eqn (1) is important at pH ≥ 8.5. The data can be fitted with eqn (2), as shown in Fig. 10, with reasonable values of the reorganization energy and pre-exponential factor. *k*_YO^−^_ is a second order rate constant that, below the diffusion-controlled limit, is equal to the product of the equilibrium constant for encounter complex formation with the oxidant (*K*_d_) and the rate constant for unimolecular ET in the encounter complex. It is reasonable to assume that *K*_d_ is constant in the present series of reactions, and it is often assumed that *K*_d_ ∼1. This means that the second-order rate constant is also expected to follow the free-energy dependence of eq. (2).^49^ The good agreement of PTET data and predictions of eqn (2) in Fig. 10 shows that the series of proteins and oxidants form a homogeneous series, where factors that may affect the ET rate constant other than the reaction free energy – such as *λ* or *K*_d_ – remain comparatively constant. Thus, the differences of the PCET rate constants between the proteins discussed in the previous paragraph can be assigned to the PT part of the reaction.

To conclude this section, the free-energy dependencies of both the CEPT and PTET rate constants show that our mechanistic assignments are consistent with current theories. The dependence of the PCET mechanism on oxidant strength and phenolic p*K*_a_ can be utilized to analyze and control the mechanism in a rational and predictable way.

## Conclusions

2MP-α_3_C and 4MP-α_3_C were designed with the specific aim of studying the effect of different degrees of solvent exposure on X_32_ properties and PCET reactivity. Both proteins could be oxidized by a series of external [Ru(L)_3_]^3+^ complexes with different oxidant strengths (*E*° = +1100–1540 mV *vs.* NHE), and a long-lived neutral radical (*t*_1/2_ > 100 s) was observed. The much greater SASA for the phenol OH of 4MP-α_3_C (30–40% *vs.* ≤∼2% for 2MP-α_3_C) did not result in any detectable increase in the rate constant for radical formation, however, and did not allow for access of buffer species as primary proton acceptors. A glutamate (E_13_) was found nearby the phenol O of 2MP-C_32_ in the solution NMR structure (O–O distance 3.2 ± 0.5 Å) and also in QM/MM MD simulations, which led to the expectation of a facilitated PCET by H-bonding and PT to E_13_. No kinetic evidence for such an effect was detected, and a variant where E_13_ was replaced with alanine (2MP-α_3_C-E_13_A) showed very similar kinetics, with even slightly faster rates. This suggests that the distance and orientation of E_13_ relative to X_32_ are not sufficiently favorable to facilitate proton tunneling, leaving water as the primary proton acceptor for all MP-α_3_C proteins.

Instead, we found that the differences in *E*°′ and p*K*_a_ values of 2MP-C_32_, 4MP-C_32_, and Y_32_ induced important changes in the rate constants and mechanisms for PCET. With the two weakest [Ru(L)_3_]^3+^ oxidants, all three MP-α_3_C proteins reacted predominantly by PTET_pre-eq_ at pH ≳ 6, with CEPT being important only at the lowest pH-values examined. This was different from α_3_Y, for which CEPT was prominent over a larger pH range and PTET_pre-eq_ dominated only at pH ≳ 8. When the strongest oxidant was used, 2MP-α_3_C and 4MP-α_3_C showed a similar balance of the two PCET mechanisms as for α_3_Y with the weaker oxidant. This can be rationalized by the lower p*K*_a_ values for the MP-α_3_C proteins, which favor PTET, but with a stronger oxidant the balance is again in favor of CEPT at neutral and acidic pH. Changing *E*°′ and p*K*_a_ values alters 
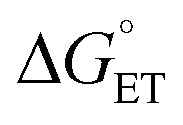
 and 
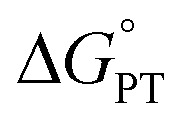
, and consequently controls which mechanism dominates the reaction, as is illustrated by the zone diagram reproduced in Fig. 9.

While the difference when changing the oxidant for a given protein can be explained by just changing the driving force (eqn (2)), the difference in *k*_CEPT_ among the proteins clearly depends also on other factors. Y_32_ has the least favorable 
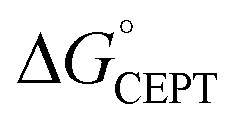
, and yet the CEPT rate constant with [Ru(bpy)_3_]^3+^ is the largest. Thus, while the three proteins appear to form a homogeneous series when comparing the oxidation rate constants for the deprotonated form (*k*_YO^−^_), the CEPT reactions differ by more than just their driving forces. MD simulations on the α_3_Y and MP-α_3_C solution NMR structures revealed fast side chain motions that allow water in and out of the X_32_ site. The observed difference in *k*_CEPT_ suggests that α_3_Y can transiently line up the phenol OH/water H-bond more favorably with respect to PT relative to the two other proteins, giving rise to the somewhat faster *k*_CEPT_ rate constant.

The present results demonstrate how the PCET mechanism for X_32_ oxidation depends on the driving forces for ET and PT. A sufficiently strong oxidant will favor ETPT and a sufficiently strong base will favor PTET, but if the driving forces for ET and PT are balanced, a concerted CEPT mechanism can dominate. This has implications for enzymes, where the p*K*_a_ of residues and reduction potentials can be altered depending on the protein environment. The mechanism in turn determines the rate of the PCET reaction and its dependence on reaction conditions. Our results also show that water is a viable proton acceptor even for amino acids with minimal solvent exposure.

## Data availability

Computational data have been deposited in the Open Science Framework Repository (DOI: https://doi.org/10.17605/OSF.IO/QPFSH).

## Author contributions

R. L. and M. L. generated purified MP-α_3_C material for the optical spectroscopy studies. A. N.-M. prepared samples, performed and analyzed all optical spectroscopy measurements. M. L. and C. T. deposited the 4MP-α_3_C NMR data and structural coordinates. C. T. developed the overall MP-α_3_C protein design strategy and performed protein structural analyses. C. R. R. performed and analyzed all computational work, under supervision of S. H.-S. P. H. performed and analyzed the EPR experiments. H. A. prepared the oxidant complexes. A. N.-M., S. D. G. S. H.-S., C. T. and L. H. conceived the study, and together with C. R. R. they prepared the manuscript draft and finalized the paper. All authors agreed on the final version of the paper.

## Conflicts of interest

There are no conflicts to declare.

## Supplementary Material

SC-015-D3SC05450K-s001
