# Supplementary material for: Switching the proton-coupled electron transfer mechanism for non-canonical tyrosine residues in a de novo protein
Source: Chem Sci. 2024 Jan 25;15(11):3957–70. doi: 10.1039/d3sc05450k (PMC10935721; doi:10.1039/d3sc05450k)
Supplement: SC-015-D3SC05450K-s001 [file SC-015-D3SC05450K-s001.pdf]

Electronic Supplementary Material (ESI) for Chemical Science.
This journal is © The Royal Society of Chemistry 2024

SUPPORTING INFORMATION TO

Switching the Proton-Coupled Electron Transfer Mechanism for Non-Canonical Tyrosine Residues in a *de novo* Protein

Astrid Nilsen-Moe[a], Clorice R. Reinhardt[b], Ping Huang[a], Hemlata Agarwala[c], Rosana Lopes[d], Mauricio Lasagna[d], Starla Glover[a], Sharon Hammes-Schiffer[e], Cecilia Tommos[d]*, Leif Hammarström[a]*

[a]Department of Chemistry, Ångström Laboratory, Uppsala University, Box 523, 75120 Uppsala, Sweden

[b]Department of Molecular Biophysics and Biochemistry, Yale University, New Haven, CT 06520, United States

[c]Technical University Munich, Campus Straubing for Biotechnology and Sustainability, Uferstraße 53, 94315 Straubing, Germany

[d]Department of Biochemistry and Biophysics, Texas A&M University, College Station, TX 77843, United States

[e]Department of Chemistry, Yale University, New Haven, CT 06520, United States

Contents:

pK_a Determinations for 2MP–α_3C and E13A variant 2
$[Ru(bpy)_3]^{2+}$ / $[Co(NH_3)_5Cl]^{2+}$ system 3
$[Ru(deeb)_3]^{2+}$ / persulfate system 7
2MP–α_3C–E13A 9
Excluding OH^- as Primary Proton Acceptor 12
Solution NMR structure of 4MP–α_3C 13
Calculation of Spin Density and Mulliken Spin Populations 13
Investigation of Donor-Acceptor Distances Between 4MP-α_3C, 2MP-α_3C, and α_3Y Side Chain Analogs and Water 18
MD Simulations with AMBER ff14SB force field 20
Additional MD Simulations Probing 2MP-E13 Hydrogen Bond 31

pK_a Determinations for 2MP–α$_3$C and E13A variant

pH titrations were performed using a Cary 5000 UV-Vis spectrophotometer and a 1×1 cm quartz cuvette. Equal volume titrations were conducted by mixing a high pH solution with a low pH solution, each containing the same [protein], and 20 mM phosphate buffer. The pH was measured before and after each recorded spectrum. The pK_a was obtained by fitting the ΔOD [298 nm (2MP–α$_3$C abs maximum) – 400 nm (baseline)] vs. pH plot to a single pK_a using Equation S1.

$$A = \frac{A_{max} + A_{min} \times 10^{pKa-pH}}{1+10^{pKa-pH}} \quad \text{(S1)}$$

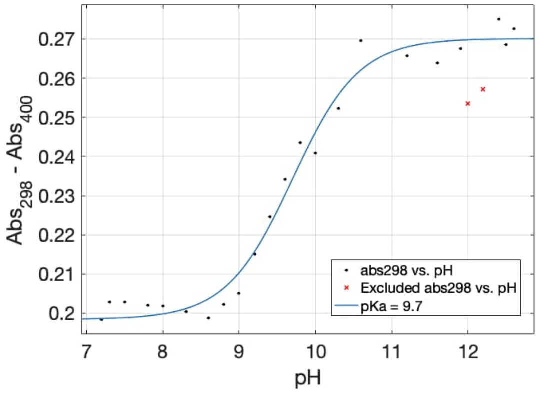

Figure S1. *Abs$_{298}$ – Abs$_{400}$ as a function of pH for 2MP–α$_3$C*. The figure shows data in black and the corresponding fit in blue. The fit yielded a pK_a of 9.7(±0.1).

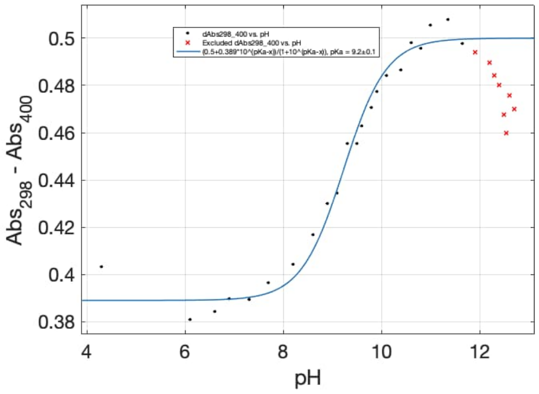

Figure S2. *Abs$_{298}$ – Abs$_{400}$ as a function of pH for 2MP–α$_3$C–E13A*. The figure shows data in black and the corresponding fit in blue. The fit yielded a pK_a of 9.2(±0.1).

$[Ru(bpy)_3]^{2+}$ / $[Co(NH_3)_5Cl]^{2+}$ system

At low pH (≤ 7), both 2MP–α_3C and 4MP–α_3C give rise to kinetic traces that changed over the first laser flashes (Figure S3), similar to what was observed for α_3Y at all pH values.[1, 2] At higher pH values (> 7), the kinetic traces were unchanged by number of laser flashes. Because of this issue, only traces after shot 10 were used in analysis.

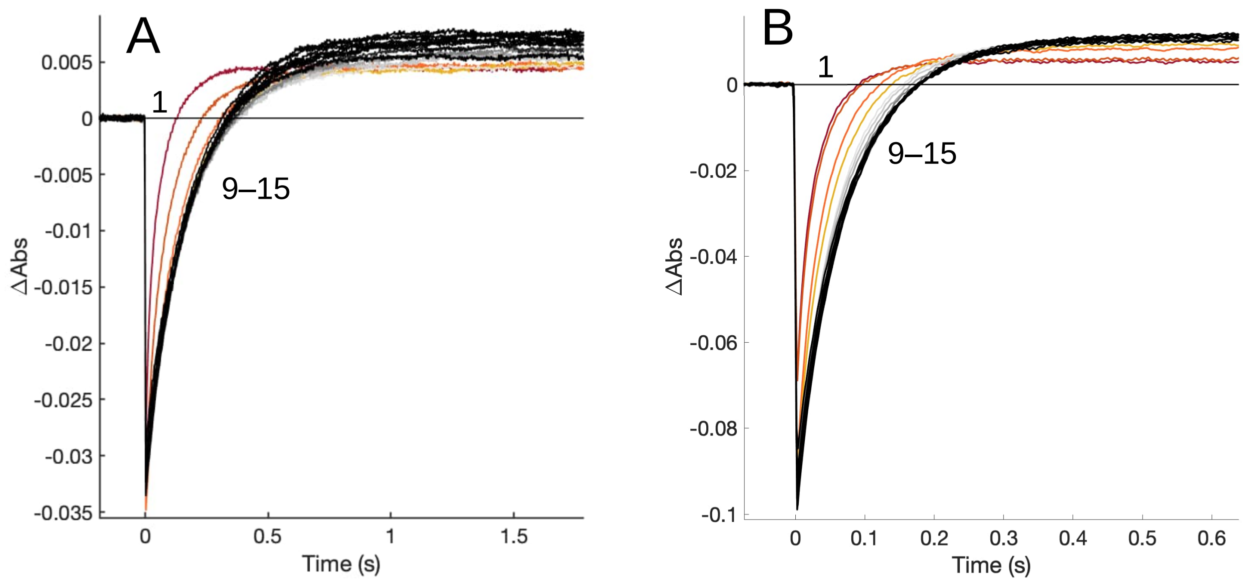

Figure S3. *Representations of the first 15 laser flash photolysis traces collected for (A) 2MP–α_3C and (B) 4MP–α_3C at low pH*. Sample conditions: 510 µM 2MP–α_3C, 30 µM $[Ru(bpy)_3]^{2+}$, 4 mM $[Co(NH_3)_5Cl]^{2+}$, 100 mM KPi, 40 mM KCl, pH 5.3(±0.2), and 540 µM 4MP–α_3C, 30 µM $[Ru(bpy)_3]^{2+}$, 6 mM $[Co(NH_3)_5Cl]^{2+}$, 100 mM KPi, 40 mM KCl, pH 5.5(±0.1).

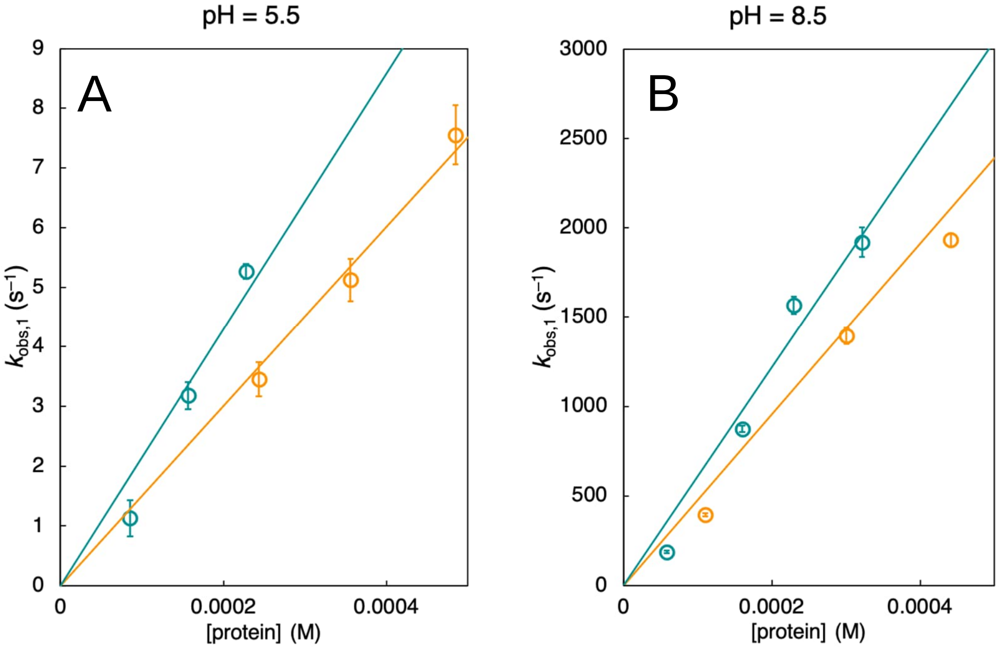

Figure S4. *Pseudo-first order rate constants, k_{obs1}, for 2MP–α_3C (orange) and 4MP–α_3C (teal) at (A) pH 5.5(±0.1) and (B) pH 8.5(±0.1) as a function of protein concentration*. Sample conditions: 30 µM

$[Ru(bpy)_3]^{2+}$, 4–5 mM $[Co(NH_3)_5Cl]^{2+}$, 100 mM KP_i, and 40 mM KCl. Linear fits were fixed to an intercept of 0. (A) Fitting the pH 5.5(±0.1) data yielded second order rate constants, k_{PCET}, of (1.5±0.04) × 10^4 $M^{-1}s^{-1}$ for 2MP–α_3C and (2.1±0.2) × 10^4 $M^{-1}s^{-1}$ for 4MP–α_3C. (B) Fitting the pH 8.5(±0.1) data yielded a k_{PCET} of (4.8±0.2) × 10^6 $M^{-1}s^{-1}$ for 2MP–α_3C and (6.1±0.4) × 10^6 $M^{-1}s^{-1}$ for 4MP–α_3C.

Table S1. *The table headings list the mean pH value (±var) calculated as an average between the pH values recorded before and after TA, the 2MP–α3C concentration used for each specific measurement, observed pseudo-first order rate constants, and calculated second order rate constants (±std).* Sample conditions: 30 μM $[Ru(bpy)_3]^{2+}$, 4–6 mM $[Co(NH_3)_5Cl]^{2+}$, 100 mM KP_i and 40 mM KCl.

pL	[2MP–α_3C] μM	k_{obs} (s^{-1})	k_{pcet} ($M^{-1}s^{-1}$)
pH = 5.3(±0.2)	240	1.3	5.4(±0.8)×10^3
pH = 5.8(±0.05)	240	2.4	9.8(±1)×10^3
pH = 6.3(±0)	220	6.3	2.8(±0.2)×10^4
pH = 7.6(±0.15)	220	9.0	4.0(±1)×10^5
pH = 7.1(±0)	620	110	1.8(±0.04)×10^5
pH = 7.8(±0.05)	620	460	7.3(±0.2)×10^5
pH = 8.6(±0.05)	310	880	2.8(±0.2)×10^6
pH = 8.9(±0.1)	620	2700	4.3(±0.2)×10^6
pD = 6.0(±0.05)	390	2.7(±0.5)	7.0(±1)×10^3
pD = 9.0(±0.05)	390	700(±30)	1.8(±0.08)×10^6

Table S2. *The table headings list the mean pH value (±var) calculated as an average between the pH values recorded before and after TA, the 4MP–α3C concentration used for each specific measurement, observed pseudo-first order rate constants, and calculated second order rate constants (±std).* Sample conditions: 30 μM $[Ru(bpy)_3]^{2+}$, 4–6 mM $[Co(NH_3)_5Cl]^{2+}$, 100 mM KP_i and 40 mM KCl.

pL	[4MP–α_3C] μM	k_{obs} (s^{-1})	k_{pcet} ($M^{-1}s^{-1}$)
pH = 5.4(±0.05)	390	6.6(±0.4)	1.7(±0.1)×10^4
pH = 5.8(±0.05)	390	11(±0.7)	2.8(±0.2)×10^4
pH = 8.8(±0.2)	370	3000(±150)	8.2(±0.4)×10^6
pH = 7.0(±0.05)	380	71(±3)	1.9(±0.08)×10^5
pH = 7.6(±0.05)	370	258(±6)	6.9(±0.2)×10^5
pH = 5.6(±0.05)	540	13	2.3(±0.4)×10^4
pH = 5.1(±0.1)	540	6.3(±0.4)	1.2(±0.02)×10^4
pH = 9.1(±0.05)	170	7000	4.2(±0.02)×10^7
pD = 5.3(±0.3)	540	1.1(±0.08)	2.0(±0.2)×10^3
pD = 6.3(±0.1)	540	4.2(±0.1)	7.8(±0.02)×10^3
pD = 8.6(±0.05)	540	320(±5)	6.0(±0.08)×10^5

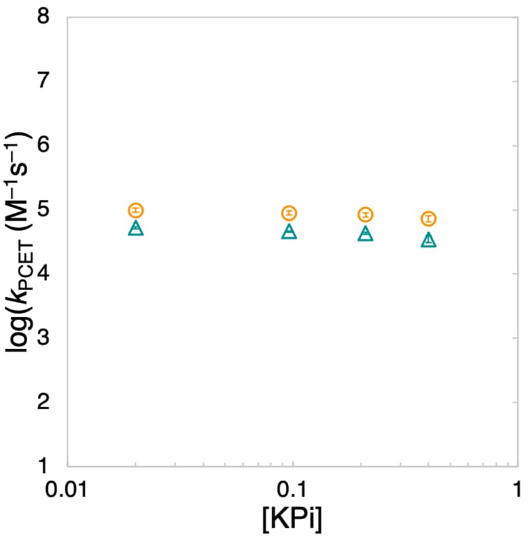

Figure S5. *Log(k_{PCET}) for the oxidation of 2MP–α_3C(orange circles) and 4MP–α_3C (teal triangles) vs. the buffer concentration*. Sample conditions: 340–360 μM protein, 25 μM $[Ru(bpy)_3]^{2+}$, 4–5 mM $[Co(NH_3)_5Cl]^{2+}$, and pH 6.5.

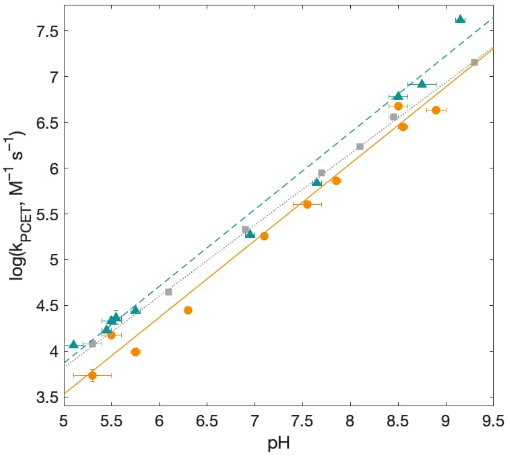

Figure S6. *Log(k_{PCET}) for the oxidation of 2MP–α_3C (orange circles with a solid line fit), 4MP–α_3C (teal triangles with a dashed line fit) and 2MP–α_3C–E13A (gray squares with a dotted line fit) vs. the pH*. The fits are to a straight line and yielded a slope of 0.84 for 2MP–α_3C and 4MP–α_3C, and 0.78 for 2MP–α_3C–E13A. Sample conditions: See Tables S1, S2, and S5.

$[Ru(dmb)_3]^{2+}$ / $[Co(NH_3)_5Cl]^{2+}$ system

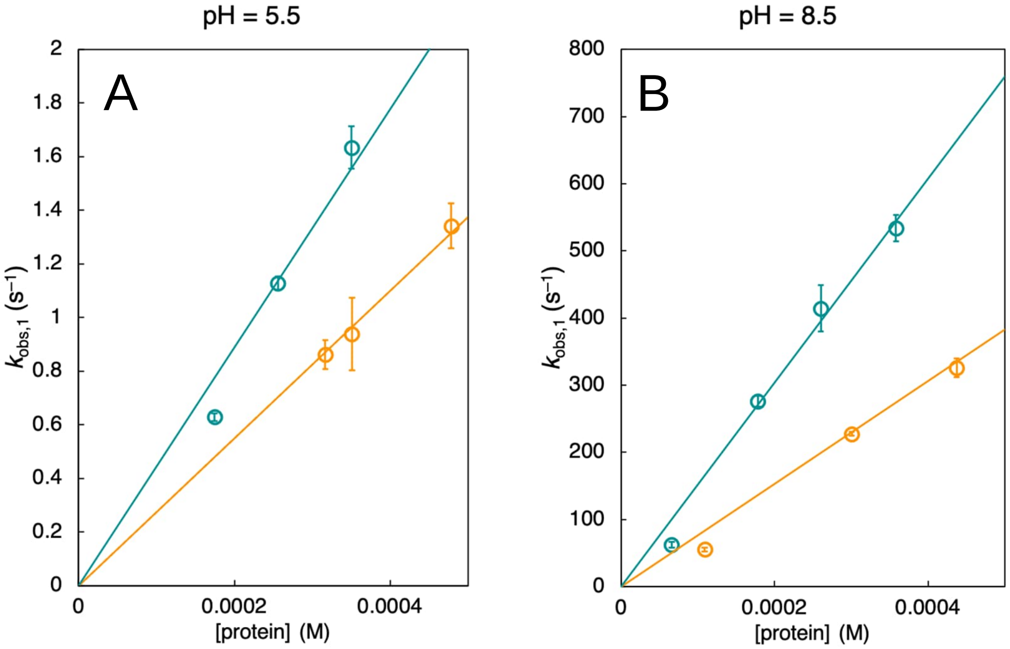

Figure S7. *Pseudo-first order rate constants, k_{obs1}, for 2MP–α_3C (orange) and 4MP–α_3C (teal) at (A) pH 5.5(±0.1) and (B) pH 8.5(±0.1) as a function of protein concentration.* Sample conditions: 20–30 µM $[Ru(dmb)_3]^{2+}$, 4–5 mM $[Co(NH_3)_5Cl]^{2+}$, 100 mM KP_i, and 40 mM KCl. Linear fits were fixed to an intercept of 0. (A) Fitting the pH 5.5(±0.1) data yielded second order rate constants, k_{PCET}, of (2.8±0.04) × 10^3 $M^{-1}s^{-1}$ for 2MP–α_3C and (4.4±0.2) × 10^3 $M^{-1}s^{-1}$ for 4MP–α_3C. (B) Fitting the pH 8.5(±0.1) data yielded a k_{PCET} of (7.7±0.2) × 10^5 $M^{-1}s^{-1}$ for 2MP–α_3C and (1.5±0.05) × 10^6 $M^{-1}s^{-1}$ for 4MP–α_3C.

$[Ru(deeb)_3]^{2+}$ / persulfate system

E°($[Ru(deeb)_3]^{2+/3+}$) determined in acetonitrile vs. the non-aqueous Ag/$AgNO_3$ reference electrode was compared to values determined for $[Ru(bpy)_3]^{2+/3+}$ under the same conditions. We assume that the difference between *E*°($[Ru(deeb)_3]^{2+/3+}$) and *E*°($[Ru(bpy)_3]^{2+/3+}$) is the same in aqueous buffer as in acetonitrile. Based on this assumption, we estimate *E*°($[Ru(dceb)_3]^{2+/3+}$) vs. the normal hydrogen electrode (NHE) using the literature *E*°($[Ru(bpy)_3]^{2+/3+}$) vs. NHE value as reference.

Using persulfate as a quencher comes with a few challenges. Because persulfate is an excellent electron acceptor, probe light photochemistry is more problematic than with $[Co(NH_3)_5Cl]^{2+}$. After about ten laser flashes to the sample, the positive amplitude from the radical species dropped significantly, indicating that significant amounts of protein had been consumed, Figure S8. Because of this, the first 3-10 shots were used in analysis.
The traces were fit with double exponential equations and only the fast component was used. The fast component represents the ET reaction between the oxidant and the protein, which is coupled to PT. The slower component represents what is likely a mix between different slower reactions such as $[Ru(deeb)_3]^{3+}$ reaction with water and $[Ru(deeb)_3]^{3+}$. It is also possible that the slow component comes from $[Ru(deeb)_3]^{3+}$ formation by reaction with the probe light, which then reacts with any of the above-described reactants as well as the protein.

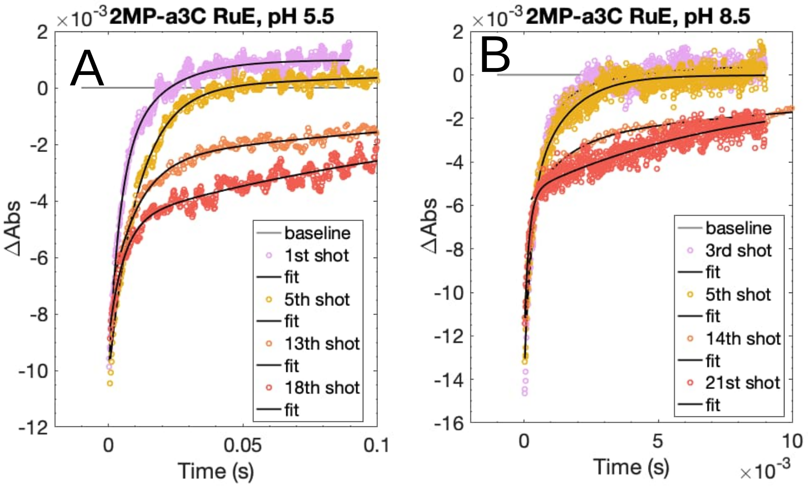

Figure S8. *2MP–C32 oxidation kinetics at pH 5.5 and 8.5 as a function of laser flashes to the sample.* TA kinetic traces after laser flash excitation were recorded at 410 nm. Sample conditions: 90 µM 2MP–α_3C, 20–30 µM $[Ru(deeb)_3]^{2+}$, 5 mM persulfate, 100 mM KP_i, and 40 mM KCl. Double exponential fits to the data are shown in black.

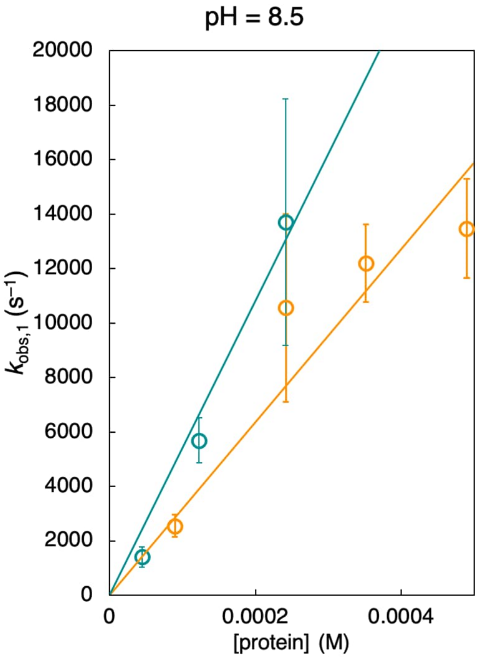

Figure S9. *Pseudo-first order rate constants, k_{obs1}, for 2MP–α_3C (orange) and 4MP–α_3C (green) at pH 8.5(±0.1) as a function of protein concentration.* Sample conditions: 20–30 μM $[Ru(deeb)_3]^{2+}$, 5 mM persulfate, 100 mM KP_i, and 40 mM KCl. Linear fits were fixed to an intercept of 0. Fits yielded second-order rate constant, k_{PCET}, of (3.2±0.3) × 10^7 $M^{-1}s^{-1}$ for 2MP–α_3C and (4.5±0.9) × 10^7 $M^{-1}s^{-1}$ for 4MP–α_3C.

Table S3. *The table headings list the mean pH value (±var) calculated as an average between the pH values recorded before and after TA, the 2MP–α_3C concentration used for each specific measurement, observed pseudo-first order rate constants, and calculated second order rate constants (±std).* Sample conditions: 20-30 μM $[Ru(dceb)_3]^{2+}$, 5 mM persulfate,100 mM KP_i and 40 mM KCl.

pL	[2MP–α_3C] μM	kobs (s^{-1})	kpcet ($M^{-1}s^{-1}$)
pH = 5.1(±0)	260	190	7.4(±0.9)×10^5
pH = 6.0(±0)	260	540	9.2(±1)×10^5
pH = 7.1(±0)	270	580	1.8(±0.3)×10^6
pH = 8.0(±0.05)	270	2800	6.3(±2)×10^6
pH = 8.8(±0.05)	270	6000	2.2(±0.3)×10^7
pD = 5.6(±0)	550	200	3.7(±0.4)×10^5

Table S4. *The table headings list the mean pH value (±var) calculated as an average between the pH values recorded before and after TA, the 4MP–α_3C concentration used for each specific measurement, observed pseudo-first order rate constants, and calculated second order rate constants (±std).* Sample conditions: 20-30 μM $[Ru(dceb)_3]^{2+}$, 5 mM persulfate, 100 mM KP_i and 40 mM KCl.

pL	[2MP–α_3C] μM	kobs (s^{-1})	kpcet ($M^{-1}s^{-1}$)
pH = 5.0(±0)	260	660(±90)	2.6(±0.3)×10^6
pH = 6.6(±0)	260	870(±100)	3.4(±0.4)×10^6
pH = 7.1(±0)	260	1000(±70)	3.9(±0.3)×10^6
pH = 9.0(±0.1)	260	25000(±700)	9.6(±3)×10^7
pD = 5.6(±0.1)	200	380(±80)	1.8(±0.4)×10^6

2MP–α$_3$C–E13A

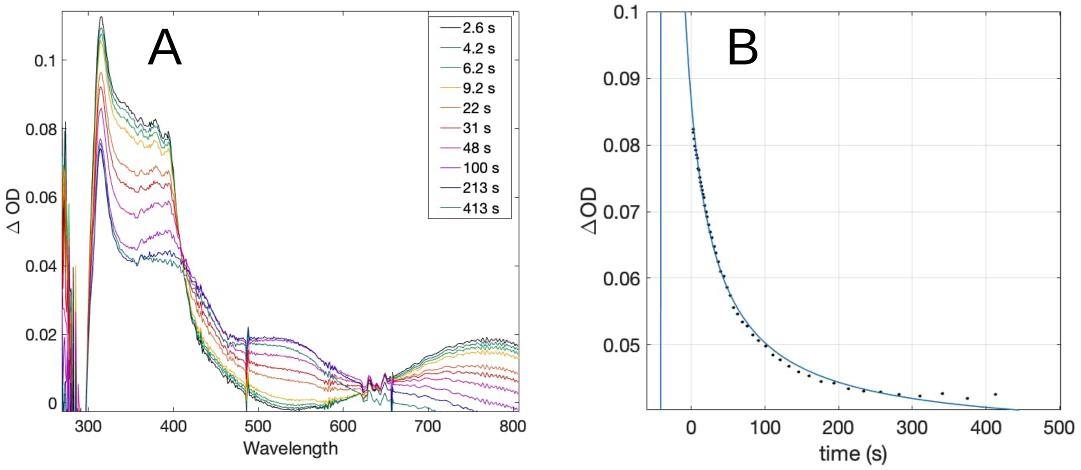

Figure S10. *Radical absorption spectra and decay kinetics traces for 2MP–α$_3$C–E13A.* (A) TA difference spectra recorded at different times following a 500 ms 447.5 nm LED pulse. (B) 2MP•–α$_3$C–E13A decay kinetics monitored at 380 nm, blue line shows a second order fit. Sample conditions: 180 μM 2MP–α$_3$C–E13A, 30 μM $[Ru(bpy)_3]^{2+}$, 3.5 mM $[Co(NH_3)_5Cl]^{2+}$, 100 mM KP_i, 40 mM KCl, pH 6.5.

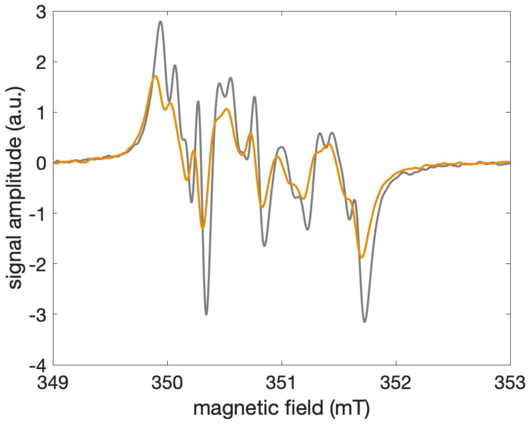

Figure S11. *EPR spectra of 2MP–α$_3$C (orange) and 2MP–α$_3$C–E13A (gray) collected at ambient temperature under continuous illumination using a 447.5 nm LED.* Sample conditions: 230-250 μM protein, 20-30 μM $[Ru(bpy)_3]^{2+}$, 4.5 mM $[Co(NH_3)_5Cl]^{2+}$, 100 mM KPi, 40 mM KCl and pH 6.5.

Table S5. *The table headings list the mean pH value (±var) calculated as an average between the pH values recorded before and after TA, the 2MP–α₃C–E13A concentration used for each specific measurement, observed pseudo-first order rate constants, and calculated second order rate constants (±std).* Sample conditions: 30 μM $[Ru(bpy)_3]^{2+}$, 4–6 mM $[Co(NH_3)_5Cl]^{2+}$, 100 mM KP_i and 40 mM KCl.

pH	[2MP–α_3C–E13A] μM	kobs (s^{-1})	kpcet ($M^{-1}s^{-1}$)
pH = 5.3(±0.1)	330	3.9(±0.3)	1.2(±0.09)×10^4
pH = 6.1(±0)	330	15(±0.6)	4.5(±0.2)×10^4
pH = 6.9(±0)	330	70(±1)	2.1(±0.04)×10^5
pH = 7.7(±0)	330	300(±7)	9.0(±0.2)×10^5
pH = 8.1(±0)	330	580(±8)	1.8(±0.02)×10^6
pH = 8.4(±0.05)	330	1200(±30)	3.6(±0.09)×10^6
pH = 9.3(±0)	330	4800(±150)	1.5(±0.04)×10^7

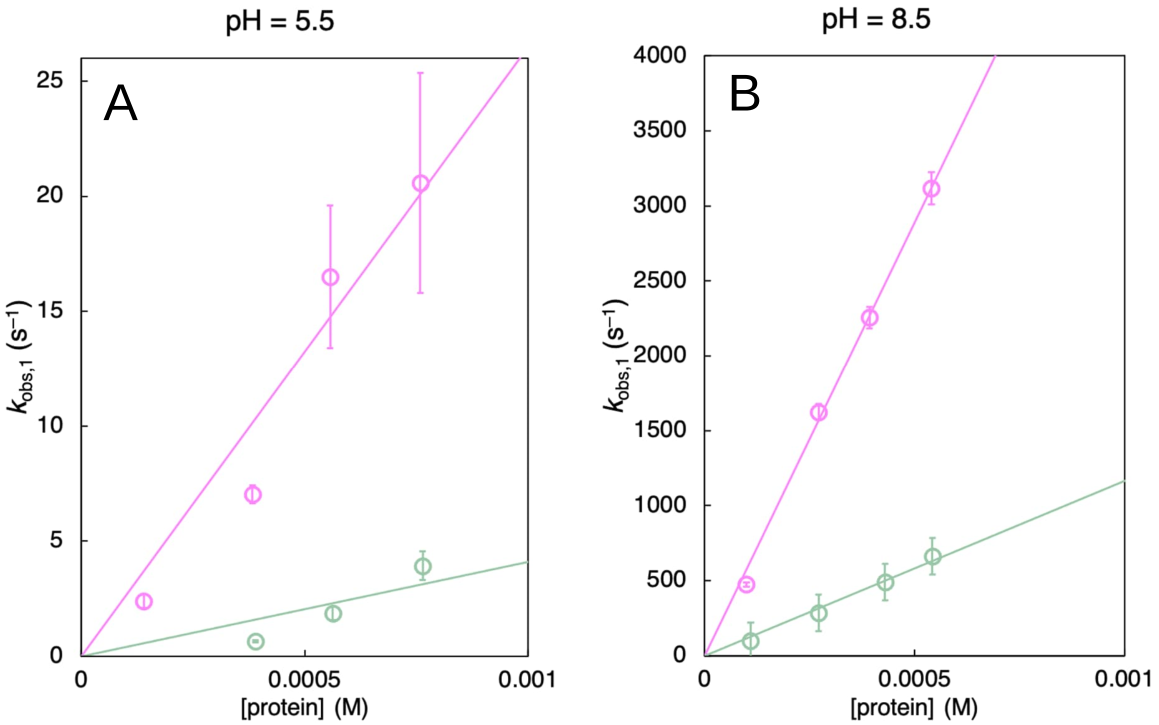

Figure S12. *Pseudo-first order rate constants, k_{obs1}, for E13A 2MP–α₃C using $[Ru(bpy)_3]^{2+}$ (pink) or $[Ru(dmb)_3]^{2+}$ (green) at (A) pH 5.5(±0.1) and (B) pH 8.5(±0.1) as a function of protein concentration.* Samples contained 30 μM $[Ru(bpy)_3]^{2+}$ or 20-30 μM $[Ru(dmb)_3]^{2+}$ and 4–5 mM $[Co(NH_3)_5Cl]^{2+}$, 100 mM KP_i, and 40 mM KCl. Linear fits were fixed to an intercept of 0. (A) Fitting the pH 5.5(±0.1) data yielded second order rate constants, k_{PCET}, of (2.6±0.2) × 10^4 $M^{-1}s^{-1}$ with $[Ru(bpy)_3]^{2+}$ as oxidant and (4.1±0.9) × 10^3 $M^{-1}s^{-1}$ with $[Ru(dmb)_3]^{2+}$ as oxidant. (B) At pH 8.5(±0.1), fits yielded k_{PCET} of (5.9±0.09) × 10^6 $M^{-1}s^{-1}$ with $[Ru(bpy)_3]^{2+}$ as oxidant and (1.2±0.04) × 10^6 $M^{-1}s^{-1}$ with $[Ru(dmb)_3]^{2+}$ as oxidant.

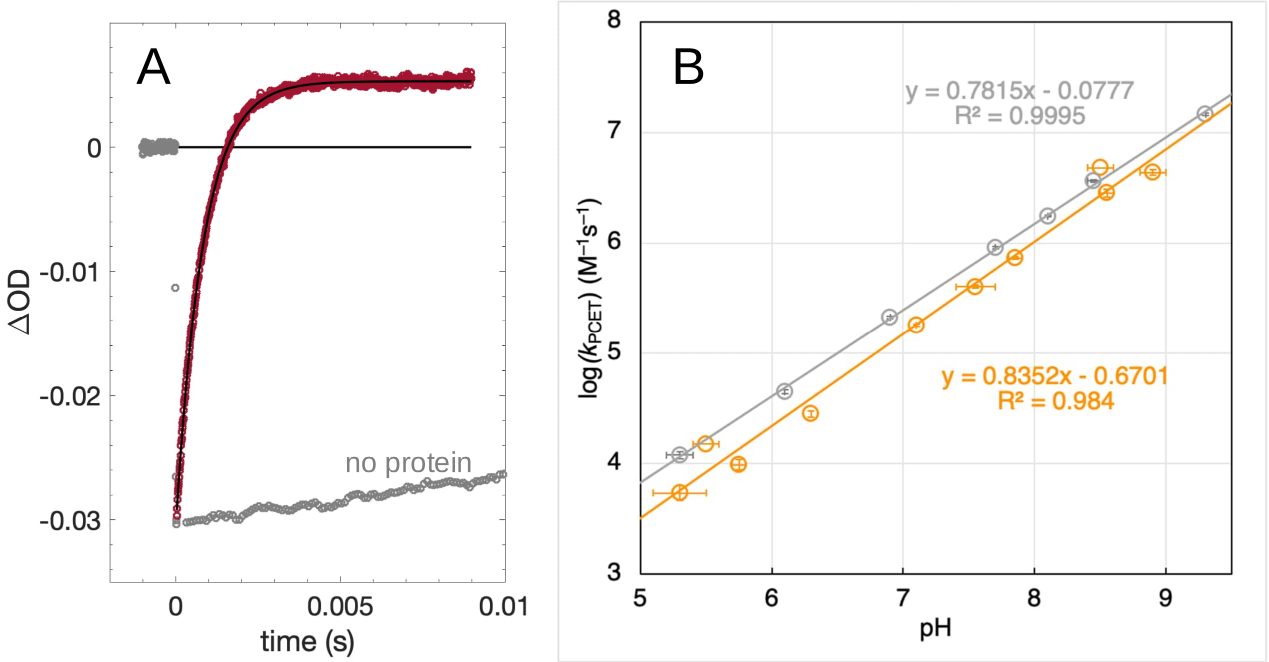

Figure S13. *(A) Representative TA trace collected at pH 8.5(±0.1) and (B) second-order rate constants reflecting the oxidation of 2MP–α$_3$C (orange) compared to E13A 2MP–α$_3$C (gray) as a function of pH.* (A) Sample conditions: 330 μM protein, 20–30 μM $[Ru(bpy)_3]^{2+}$, 4–5 mM $[Co(NH_3)_5Cl]^{2+}$, 100 mM KP_i and 40 mM KCl buffer (B) Sample conditions: 170–540 μM 2MP–α$_3$C, 330 μM 2MP–α$_3$C–E13A, 20–30 μM $[Ru(bpy)_3]^{2+}$, 4–5 mM $[Co(NH_3)_5Cl]^{2+}$, 100 mM KP_i, and 40 mM KCl. Straight lines show a linear fit to the data, with the equations shown in the plot.

Excluding OH^- as Primary Proton Acceptor

Following the same argument published in the SI of ref [3], summarized below, we exclude OH^- as primary proton acceptor for a concerted PCET mechanism when $[Ru(bpy)_3]^{3+}$ or $[Ru(deeb)_3]^{3+}$ were used as oxidants.

The concerted PCET reaction from MP–α_3C with OH^- as the primary proton acceptor can be analyzed as OH^- reacting with the [Ru(III)…MP–α_3C] encounter complex (where Ru(III) can be either $[Ru(bpy)_3]^{3+}$ or $[Ru(deeb)_3]^{3+}$):

$$\text{Ru(III)+ MP–}\alpha_3\text{C} \underset{k_{-d}}{\overset{k_d}{\rightleftharpoons}} \text{[Ru(III)…MP–}\alpha_3\text{C]} \quad \text{(S1)}$$

$$\text{[Ru(III)…MP–}\alpha_3\text{C]+ OH}^- \xrightarrow{k_{OH-}} \text{Ru(II) + MP•–}\alpha_3\text{C + H}_2\text{O} \quad \text{(S2)}$$

This reaction is sufficiently downhill such that it can be treated as irreversible. The steady-state approximation then yields the following expression:

$$\text{rate} = \frac{d[\text{MP•–}\alpha_3\text{C}]}{dt} = k_{obs}[\text{Ru(III)}][\text{MP–}\alpha_3\text{C}] \quad \text{(S3)}$$

where the pseudo-second order rate constant k_{obs} is defined as:

$$k_{obs} = \frac{k_d k_{OH-}[OH^-]}{k_{-d} + k_{OH-}[OH^-]} \quad \text{(S4)}$$

Assuming that there is no specific driving force for formation of the complex in reaction S1, i.e., $\Delta G° = 0$ we get $k_d/k_{-d} = 1\ M^{-1}$.

If we assume that the diffusional rate constant k_{diff} equals$10\times10^{10}\ M^{-1}s^{-1}$ in water, this is the maximum value that k_{OH-} can take (it is probably smaller given that the protein is large and therefore slow to diffuse). $k_{OH-}[OH^-]$ is very likely much smaller than k_{-d} which simplifies Eq. S4 to:

$$k_{obs} = \frac{k_d k_{OH-}[OH^-]}{k_{-d}} \quad \text{(S5)}$$

where $k_d/k_{-d} = 1\ M^{-1}$.

$$k_{obs} = k_{OH-}[OH^-]/M \quad \text{(S6)}$$

This allows us to compare the measured $k_{obs} = k_{PCET}$ rate constants with the $[OH^-]$. The results show that even at the highest pH-values, where the $[OH^-]$ is the largest, k_{OH-} cannot account for the rate constants: $k_{PCET} = 1.8(\pm0.08)\times10^6\ M^{-1}s^{-1}$ (for 2MP–α_3C/$[Ru(bpy)_3]^{3+}$ at pH 9.0), $[OH^-]_{(pH\ 9)} = 1\times10^{-5}$ M making $k_{OH-} > 1\times10^{11}\ M^{-1}s^{-1}$ which is faster than a diffusional controlled reaction.

While this derivation is based on several assumptions, k_{OH-} is likely much smaller than the diffusional controlled reaction rate assumed for small molecules in water ($k_{diff} = 10\times10^{10}\ M^{-1}s^{-1}$) given the size of the protein.

Solution NMR structure of 4MP–α_3C

Table S6. *Experimental solution NMR restraints and structural statistics of 4MP–α_3C and 2MP–α_3C*

Experimental NMR restraints	4MP–α_3C	2MP– α_3C[24]
NOE intra-residue	294	216
NOE sequential (|i-j| = 1)	151	222
NOE medium range (1 < |i-j| < 5)	213	181
NOE long range (|i-j| ≥ 5)	171	190
NOE total	829	809
backbone dihedral angles	108	107
hydrogen bond restraints	39	44
Experimental restraints total	976	960
average restraints per residue	15.0	14.8
number of restrained residues	65	65
Ramachandran plot summary		
most favored regions	96.6%	96.4%
additionally allowed regions	1.5%	3.2%
RMS deviations from experimental restraints		
NOE-based distances	0.0061 ± 0.0005 Å	0.0061 ± 0.0003 Å
backbone dihedral angle	0.16 ± 0.03°	0.22 ± 0.02°
RMS deviations from idealized geometry		
Bonds	0.0011 ± 0.0001 Å	0.0011 ± 0.0001 Å
Angles	0.35 ± 0.01°	0.33 ± 0.01°
Impropers	0.20 ± 0.01°	0.20 ± 0.01°
Average RMS from mean coordinates		
All residues		
backbone atoms	0.48 Å	0.46 Å
all atoms	1.21 Å	0.95 Å
Helical residues		
backbone atoms	0.30 Å	0.27 Å
all atoms	1.20 Å	0.90 Å
MP–C32	1.01 Å	0.61 Å

4MP–α_3C structures were generated from the experimental NMR restraints listed above by simulated annealing. NOE-derived proton−proton distance restraints were grouped in distance ranges of 1.7−3.0, 1.7−4.0, and 1.7−5.0 Å corresponding to strong, medium, and weak NOE cross-peak intensities, respectively. When one or two methyl groups were involved, the upper boundary was increased by 0.5 or 1.0 Å, respectively. Backbone dihedral angle and hydrogen bond restraints were derived from the secondary structure predictions made by the TALOS+. One thousand trial structures were generated and further evaluated using the CNS accept.inp script to obtain a collection of refined structures. The 32 lowest-energy structures from this collection form the deposited 4MP–α_3C structural ensemble (PDB ID 8VSW, BMRB ID 31067).

Calculation of Spin Density and Mulliken Spin Populations

The spin densities were calculated for optimized geometries of the 4MP-α_3C, 2MP-α_3C, and α_3Y side chain analogs in their neutral and cationic radical states with unrestricted DFT using Gaussian 16.[4] The spin densities were visualized, and the Mulliken spin population[5] values were computed. To examine if the trends observed depend on the DFT functional or basis set, we optimized the geometries and calculated

the spin populations with three different functionals, ωB97X-D,[6] B3LYP-D3(BJ),[7-9] and M06-2X,[10] with the 6-31G** and 6-31+G** basis sets. Additionally, the 6-31++G** basis set[11-13] was used with the ωB97X-D functional. A tight convergence criterion (Opt=Tight) was used, and the optimized geometries were confirmed to be minima because they do not have any imaginary frequencies. The values of the spin populations for the two sulfur atoms and oxygen atoms in the sidechains at these levels of theory are given in Table S7. The spin densities for the DFT calculations at the ωB97X-D/6-31G** level of theory are visualized in Figure S14.

The spin densities and spin populations were also determined for these species with the complete active space self-consistent-field (CASSCF) method.[14, 15] The geometries used for these calculations were optimized with DFT at the ωB97X-D/6-31+G** level of theory (Table S8). The active spaces were chosen with the automated π-orbital space (PiOS) method,[16] including the heavy atoms of the aromatic ring and adjacent sulfur and oxygen atoms, which resulted in a (9e, 8o) active space for the 4MP-α_3C and 2MP-α_3C sidechain models, and a (7e,7o) active space for the Y sidechain model. An aug-cc-pVTZ basis set was used for the CASSCF calculations,[17, 18] and the PySCF program was used to perform the computations.[19, 20] The spin densities for the CASSCF calculations are shown in Figure 6 in the main paper.

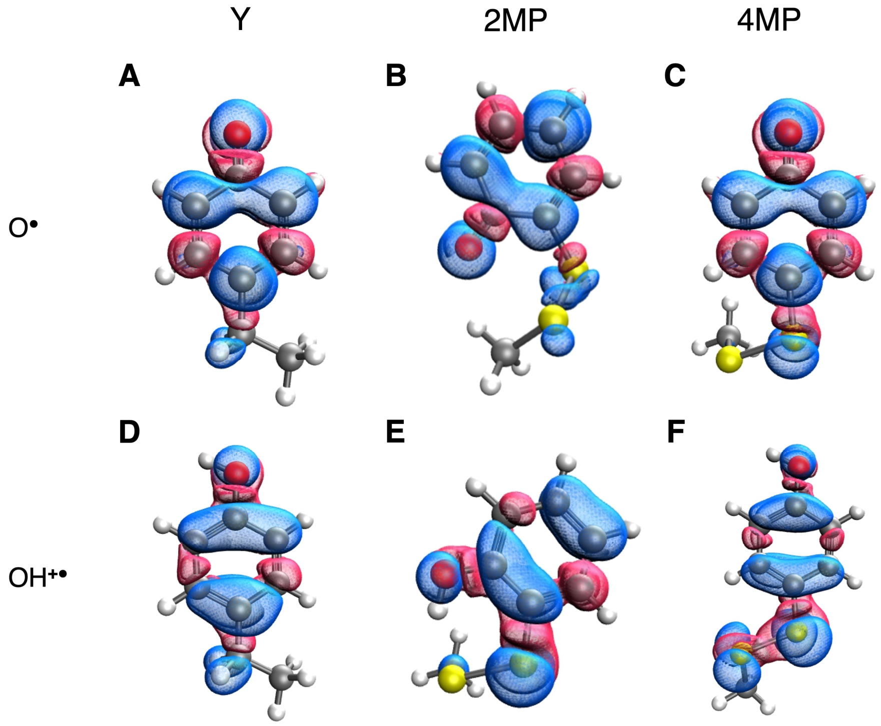

Figure S14. *Spin densities computed at the ωB97X-D/6-31G** level of theory, visualized with an isosurface value of 0.02 Å*$^{-3}$.

Table S7. *Mulliken spin populations on key atoms of side chain analogs at various levels of theory[a]*

System	O	S	S_2	Total S[b]	Level of Theory
4MPC-O•	0.399	0.067	-0.004	0.063	ωB97X-D/6-31G**
2MPC-O•	0.383	0.022	0.026	0.048	ωB97X-D/6-31G**
Y-O•	0.422	---	----	----	ωB97X-D/6-31G**
4MPC-OH+•	0.087	0.386	0.124	0.510	ωB97X-D/6-31G**
2MPC-OH+•	0.123	0.263	-0.006	0.257	ωB97X-D/6-31G**
Y-OH+•	0.165	---	----	----	ωB97X-D/6-31G**
4MPC-O•	0.374	0.089	-0.003	0.086	ωB97X-D/6-31+G**
2MPC-O•	0.361	0.035	0.024	0.059	ωB97X-D/6-31+G**
Y-O•	0.399	---	----	----	ωB97X-D/6-31+G**
4MPC-OH+•	0.073	0.419	0.146	0.565	ωB97X-D/6-31+G**
2MPC-OH+•	0.143	0.261	-0.007	0.254	ωB97X-D/6-31+G**
Y-OH+•	0.152	---	----	----	ωB97X-D/6-31+G**
4MPC-O•	0.372	0.091	-0.003	0.088	ωB97X-D/6-31++G**
2MPC-O•	0.360	0.035	0.025	0.060	ωB97X-D/6-31++G**
Y-O•	0.397	---	----	----	ωB97X-D/6-31++G**
4MPC-OH+•	0.073	0.420	0.147	0.567	ωB97X-D/6-31++G**
2MPC-OH+•	0.142	0.265	-0.008	0.257	ωB97X-D/6-31++G**
Y-OH+•	0.151	---	----	----	ωB97X-D/6-31++G**
4MPC-O•	0.398	0.086	-0.004	0.082	B3LYP-D3(BJ)/6-31G**
2MPC-O•	0.378	0.050	0.026	0.076	B3LYP-D3(BJ)/6-31G**
Y-O•	0.428	---	----	----	B3LYP-D3(BJ)/6-31G**
4MPC-OH+•	0.089	0.360	0.180	0.540	B3LYP-D3(BJ)/6-31G**
2MPC-OH+•	0.138	0.276	0.053	0.329	B3LYP-D3(BJ)/6-31G**
Y-OH+•	0.172	---	----	----	B3LYP-D3(BJ)/6-31G**
4MPC-O•	0.372	0.107	-0.002	0.105	B3LYP-D3(BJ)/6-31+G**
2MPC-O•	0.355	0.067	0.023	0.090	B3LYP-D3(BJ)/6-31+G**
Y-O•	0.404	---	----	----	B3LYP-D3(BJ)/6-31+G**
4MPC-OH+•	0.078	0.382	0.196	0.578	B3LYP-D3(BJ)/6-31+G**
2MPC-OH+•	0.112	0.325	0.084	0.409	B3LYP-D3(BJ)/6-31+G**
Y-OH+•	0.160	---	----	----	B3LYP-D3(BJ)/6-31+G**
4MPC-O•	0.402	0.077	-0.005	0.072	M062X/6-31G**
2MPC-O•	0.385	0.040	0.021	0.061	M062X/6-31G**
Y-O•	0.429	---	----	----	M062X/6-31G**
4MPC-OH+•	0.071	0.405	0.177	0.582	M062X/6-31G**
2MPC-OH+•	0.084	0.399	-0.155	0.244	M062X/6-31G**
Y-OH+•	0.168	---	----	----	M062X/6-31G**
4MPC-O•	0.377	0.097	-0.003	0.094	M062X/6-31+G**
2MPC-O•	0.366	0.051	0.019	0.070	M062X/6-31+G**
Y-O•	0.406	---	----	----	M062X/6-31+G**
4MPC-OH+•	0.071	0.405	0.177	0.582	M062X/6-31+G**
2MPC-OH+•	0.140	0.278	-0.005	0.273	M062X/6-31+G**
Y-OH+•	0.119	---	----	----	M062X/6-31+G**
4MPC-O•	0.348	0.039	0.001	0.040	CASSCF/6-31G**
2MPC-O•	0.329	0.011	0.007	0.018	CASSCF/6-31G**
Y-O•	0.365	---	----	----	CASSCF/6-31G**
4MPC-OH+•	0.053	0.401	0.067	0.467	CASSCF/6-31G**
2MPC-OH+•	0.129	0.104	0.003	0.107	CASSCF/6-31G**
Y-OH+•	0.113	---	----	----	CASSCF/6-31G**
4MPC-O•	0.323	0.044	0.002	0.046	CASSCF/aug-cc-pvdz
2MPC-O•	0.313	0.010	0.006	0.016	CASSCF/aug-cc-pvdz
Y-O•	0.341	---	----	----	CASSCF/aug-cc-pvdz
4MPC-OH+•	0.041	0.453	0.078	0.531	CASSCF/aug-cc-pvdz
2MPC-OH+•	0.117	0.102	0.006	0.108	CASSCF/aug-cc-pvdz
Y-OH+•	0.102	---	----	----	CASSCF/ aug-cc-pvdz
4MPC-O•	0.321	0.049	0.002	0.051	CASSCF/aug-cc-pvtz
2MPC-O•	0.310	0.013	0.006	0.019	CASSCF/aug-cc-pvtz
Y-O•	0.340	---	----	----	CASSCF/aug-cc-pvtz
4MPC-OH+•	0.041	0.453	0.078	0.531	CASSCF/aug-cc-pvtz
2MPC-OH+•	0.120	0.105	0.003	0.108	CASSCF/aug-cc-pvtz
Y-OH+•	0.106	---	----	----	CASSCF/aug-cc-pvtz

[a]"O" refers to the hydroxyl oxygen of the sidechain, "S" refers to the sulfur atom closest to the phenol ring, and "S_2" refers to the sulfur atom most distal from the phenol ring, i.e., closest to the backbone. [b]"Total S" refers to the total spin population on the sulfur atoms in the molecule. Some of these results are also provided in Table 2 in the main paper.

Table S8. *Geometries used for CASSCF Mulliken spin population analysis in XYZ format.* These geometries were obtained from DFT optimizations at the ωB97X-D/6-31+G** level of theory.

18

Y analog neutral radical

H	2.1155830000	-2.1125400000	0.8594190000
C	1.5857110000	-1.2513380000	0.4657190000
C	0.3376740000	-1.4998970000	-0.2339460000
O	-0.1144640000	-2.6563490000	-0.3808350000
C	-0.3575250000	-0.3390320000	-0.7535220000
H	-1.2912050000	-0.5142710000	-1.2780100000
C	0.1497250000	0.9292020000	-0.5857420000
C	1.3644620000	1.1452260000	0.0978130000
C	1.9577180000	2.5178900000	0.3048670000
C	2.0627290000	0.0232410000	0.6152940000
H	2.9991680000	0.1922320000	1.1419960000
H	-0.3969820000	1.7758920000	-0.9877230000
C	1.1730640000	3.6943630000	-0.2682340000
H	2.9703140000	2.5135820000	-0.1200170000
H	2.0973540000	2.6640680000	1.3842240000
H	1.0608130000	3.6135380000	-1.3538990000
H	1.6970970000	4.6303790000	-0.0585010000
H	0.1747690000	3.7671980000	0.1743560000

19

Y analog cationic radical

H	2.1704830000	-2.1110950000	0.8067960000
C	1.6229530000	-1.2568510000	0.4199530000
C	0.3953230000	-1.4506380000	-0.2805780000
O	-0.1277080000	-2.6316470000	-0.4922330000
C	-0.3387900000	-0.3372780000	-0.7966330000
H	-1.2668510000	-0.5337720000	-1.3222060000
C	0.1450060000	0.9279320000	-0.6146710000
C	1.3667510000	1.1488450000	0.0801050000
C	1.9365750000	2.5089920000	0.3046780000
C	2.0848740000	0.0144440000	0.5886790000
H	3.0170390000	0.1848450000	1.1186940000
H	-0.4083340000	1.7741210000	-1.0042900000
C	1.1569200000	3.6962120000	-0.2478520000
H	2.9596770000	2.4979180000	-0.1022490000
H	2.0843120000	2.6178640000	1.3904440000
H	1.0570970000	3.6461990000	-1.3358130000
H	1.6879960000	4.6198120000	-0.0117000000
H	0.1601450000	3.7703510000	0.1960550000
H	0.3960320000	-3.3613660000	-0.1292520000

17

2MP analog neutral radical

S	4.13813500	-0.21525300	-0.49085500

C	2.43318200	0.20820200	-0.28491400
C	1.40023600	-0.80646600	-0.04128400
C	0.02782800	-0.33342600	0.05015400
H	-0.72392700	-1.08667300	0.25928000
C	-0.29199600	0.98345600	-0.14089900
C	0.72455300	1.92199900	-0.41247800
H	0.46897300	2.96637600	-0.55776100
C	2.06658600	1.53041500	-0.47244700
H	2.83569000	2.27424100	-0.65400400
H	-1.32437800	1.31215900	-0.08240200
S	4.68930800	-1.20513600	1.24173800
C	4.55960200	-2.96027700	0.78834500
H	4.85399900	-3.51600500	1.68268100
H	5.24368900	-3.20081700	-0.02632400
H	3.53063100	-3.19193300	0.51828700
O	1.65580700	-2.01792900	0.06278700

18
2MP analog cation radical

S	3.85658500	-0.08589700	1.15007300
C	2.30218300	0.17713600	0.42903000
C	1.16123800	-0.72611100	0.37633600
C	-0.04096100	-0.30396100	-0.24138000
H	-0.86169200	-1.01131700	-0.26991100
C	-0.14037900	0.95571100	-0.75838100
C	0.96441000	1.85861800	-0.69107100
H	0.86738700	2.85457500	-1.10837300
C	2.14675600	1.47677000	-0.11103500
H	2.98208900	2.16900000	-0.07362400
H	-1.06477900	1.27897400	-1.22409400
S	4.04324600	-2.07995900	1.71070600
C	4.76700600	-2.83405400	0.21533300
H	4.94230400	-3.87773500	0.48876900
H	5.71937700	-2.36023100	-0.02190900
H	4.08023500	-2.78800800	-0.62994200
O	1.15885300	-1.93526800	0.87125100
H	2.01502500	-2.19346100	1.28953200

17
4MP analog neutral radical

S	4.2172440000	0.1125700000	0.6274010000
C	2.4914000000	0.2988210000	0.2905640000
C	1.4809670000	-0.5492470000	0.7922370000
C	0.1666510000	-0.3174820000	0.4750850000
H	-0.6254820000	-0.9562260000	0.8516750000
C	-0.2255090000	0.7971150000	-0.3694380000
C	0.8452440000	1.6515650000	-0.8555280000
H	0.5665040000	2.4888640000	-1.4864790000
C	2.1516680000	1.4027030000	-0.5337150000

H	2.9392720000	2.0478680000	-0.9139220000
O	-1.4192200000	1.0096820000	-0.6624760000
S	4.4188940000	-1.6846760000	1.6129820000
C	4.3844440000	-2.8740930000	0.2358940000
H	4.5374900000	-3.8595950000	0.6831100000
H	5.1879600000	-2.6692320000	-0.4726810000
H	3.4177200000	-2.8558730000	-0.2708480000
H	1.7526730000	-1.3798330000	1.4360400000

18
4MP analog cationic radical

S	3.7848940000	-0.5450110000	0.0050310000
C	2.1644680000	0.0288200000	-0.0324600000
C	1.0291950000	-0.7063270000	0.3870390000
C	-0.2198380000	-0.1410080000	0.3065360000
H	-1.0920570000	-0.7080530000	0.6169370000
C	-0.3695060000	1.1701270000	-0.1995530000
C	0.7570930000	1.9052430000	-0.6325070000
H	0.6108710000	2.9070590000	-1.0189320000
C	2.0039510000	1.3457230000	-0.5489520000
H	2.8714880000	1.9149290000	-0.8684480000
O	-1.5435400000	1.7723310000	-0.3065080000
S	3.7533740000	-2.1842000000	1.2399560000
C	5.1743510000	-3.0921780000	0.5558190000
H	5.3023120000	-3.9423190000	1.2293900000
H	6.0788050000	-2.4830120000	0.5797730000
H	4.9597360000	-3.4516340000	-0.4503240000
H	1.1336580000	-1.7211870000	0.7539740000
H	-2.2768030000	1.2275000000	0.0060170000

Investigation of Donor-Acceptor Distances Between 4MP-α_3C, 2MP-α_3C, and α_3Y Side Chain Analogs and Water

Gas phase geometries for the sidechain models hydrogen bonded to water were optimized at the DFT/B3LYP/6-311++G** level of theory, as shown in Figure S15. The geometries were confirmed to be minima because no imaginary frequencies were observed, and a tight convergence criterion (Opt=Tight) was used. Additionally, the geometries were optimized in implicit solvent using the integral equation formalism polarizable continuum model[21-23] with water as the solvent. For comparison, the calculations were also performed with the ωB97X-D functional. Distances and angles for the hydrogen bond to water are given in Table S9.

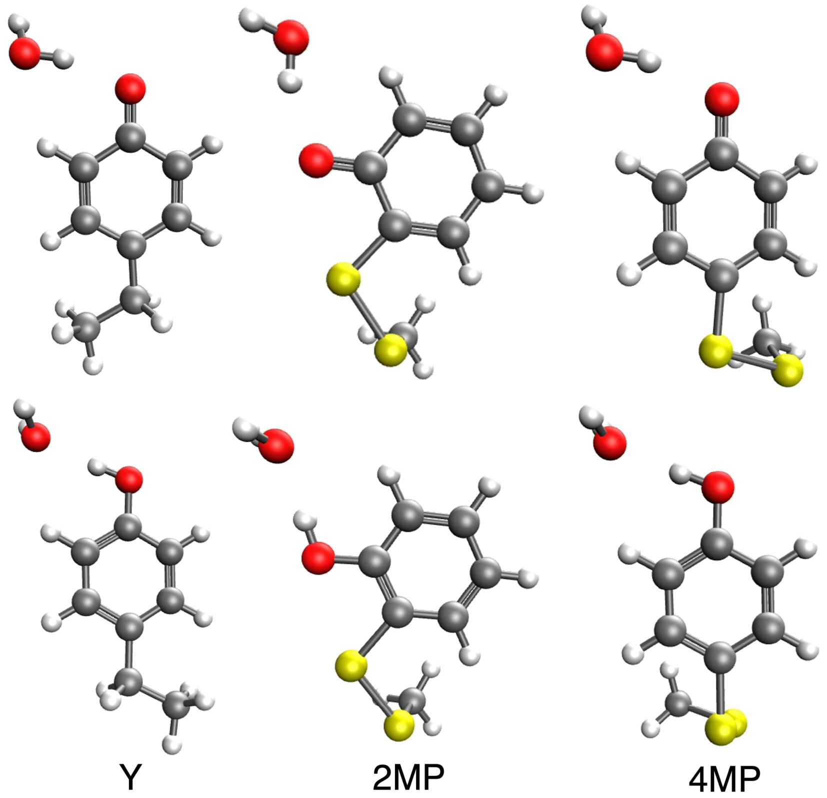

Figure S1. *DFT/B3LYP/6-311++G** optimized gas-phase geometries of neutral Y, 2MP, and 4MP radical (top) and standard OH (bottom) species hydrogen bonding to water.* Donor-acceptor distances and hydrogen bonding angles are given in Table S9.

Table S9. *Donor-acceptor distances and angles for the hydrogen bond of sidechain model to water*. These values were obtained in the gas phase except the values in parentheses were calculated with implicit solvation.

System	Functional/Basis Set	Distance (Å)	Angle (°)
4MPC-O$^{\bullet}$	B3LYP/6-311++G**	2.83 (2.80)	162.3 (178.4)
2MPC-O$^{\bullet}$	B3LYP/6-311++G**	2.84 (2.82)	161.7 (178.3)
Y-O$^{\bullet}$	B3LYP/6-311++G**	2.84 (2.81)	162.9 (178.3)
4MPC-OH	B3LYP/6-311++G**	2.83 (2.75)	175.8 (177.4)
2MPC-OH	B3LYP/6-311++G**	2.82 (2.74)	174.7 (176.6)
Y-OH	B3LYP/6-311++G**	2.85 (2.77)	174.7 (170.7)
4MPC-O$^{\bullet}$	ωB97XD/6-311++G**	2.82	160.7
2MPC-O$^{\bullet}$	ωB97XD/6-311++G**	2.83	160.2
Y-O$^{\bullet}$	ωB97XD/6-311++G**	2.83	162.0
4MPC-OH	ωB97XD/6-311++G**	2.81	178.6
2MPC-OH	ωB97XD/6-311++G**	2.80	176.7
Y-OH	ωB97XD/6-311++G**	2.83	177.9

MD Simulations with AMBER ff14SB force field

For the 2MP-α_3C[24] and 4MP-α_3C systems, two frames of the NMR ensemble were chosen randomly to use as starting points for solvation and equilibration of two independent trajectories per system (frames 3 and 28 for each system). The starting structures were solvated with TIP3P water,[25] and Cl^- ions were added to neutralize the +2 charge, and then Na^+/Cl^- ions were added to produce a salt concentration of ~150 mM.

As the 4MP and 2MP sidechains are not part of the standard protein force field, partial charges were assigned by the RESP procedure.[26] In this protocol, the geometries of the sidechains and added N-methyl and acetyl blocking groups were optimized with the Hartree-Fock method and the 6-31G* basis set.[11, 12] Two conformers were generated with φ and ψ angles corresponding to a β-sheet or corresponding to an α helix, following the precedent set by Ref. [27]. This workflow was followed to be consistent with the existing force field parameters. The atomic charges of the backbone atoms (C,O,N,H) were fixed to the corresponding charges of the canonical cysteine in the AMBER library file during the RESP fitting procedure. A group constraint on the blocking N-methyl and acetyl groups was used to force a neutral charge for the 2MP and 4MP sidechains. Partial charges and force field parameters are provided in Table S10 according to the atom naming given in Figure S16, and the optimized geometries used for the RESP procedure are given in Table S11.

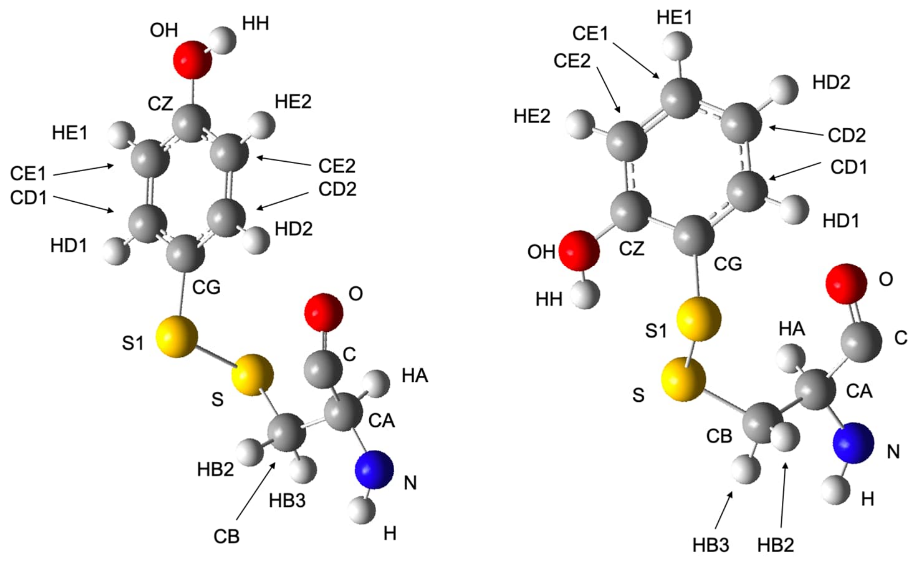

Figure S16. *Atom naming used for MP sidechains.* The naming is analogous to that for the Y and C sidechains when possible.

Table S10. *Force field parameters and partial charges for simulations containing MP sidechains*

MPC params
BOND
S - CA 317.0   1.74 ! kb from CA-2C, equil distance from 2LXY structure

ANGL
S -S -CA   68.0 103.7 ! taken from 2C-S -S, 68.0 103.7, 2LXY value is 103.64 .
S -CA -CA   70.0 125.4 ! kb from CA-CA-2C, equil value from structure, value from CA-CA-2C is 120.0

S -CA -C   70 125.4! same as S-CA-CA for consistency
HC -2C -S 42.100 116.190 ! same as h4-ca-ss

DIHE
CA-S-S -2C   1   0.000   0.000   3.000   same as X -ss-ss-X
CA-CA-S -S   2   0.800   180.000   2.000   same as X -ca-ss-X
C -CA-S -S   2   0.800   180.000   2.000   same as X -ca-ss-X
CA-CA-CA-S   4   14.500   180.000   2.000   same as X -ca-ca-X

4MP

Atom name	Atom type	Charge	Atom name	Atom type	Charge
N	N	-0.415700	CZ	C	0.484349
H	H	0.271900	OH	OH	-0.576372
CA	CX	0.030415	HH	HO	0.402916
HA	H1	0.101741	CE2	CA	-0.419558
CB	2C	0.022254	HE2	HA	0.207024
HB2	HC	0.057568	CD2	CA	-0.029935
HB3	HC	0.057568	HD2	HA	0.144132
CG	CA	0.092698	C	C	0.597300
CD1	CA	-0.029935	O	O	-0.567900
HD1	HA	0.144132	S	S	-0.182969
CE1	CA	-0.419558	S1	S	-0.171914
HE1	HA	0.207024			

2MP

Atom name	Atom type	Charge	Atom name	Atom type	Charge
N	N	-0.415700	CZ	C	0.423893
H	H	0.271900	OH	OH	-0.586836
CA	CX	0.045748	HH	HO	0.404063
HA	H1	0.104799	CE2	CA	-0.353871
CB	2C	-0.078027	HE2	HA	0.198293
HB2	HC	0.087509	CD2	CA	-0.264055
HB3	HC	0.087509	HD2	HA	0.163280
CG	CA	0.022223	C	C	0.597300
CD1	CA	-0.107076	O	O	-0.567900
HD1	HA	0.193249	S	S	-0.158133
CE1	CA	-0.054109	S1	S	-0.160445
HE1	HA	0.146386			

Table S11. *Cartesian coordinates in xyz file format of geometries used for RESP fitting of partial charges*

35

4MP-alpha helical conformation

H	-6.53840	0.76790	1.12605
C	-5.92497	-0.10682	0.96739
H	-6.23308	-0.61072	0.05799
H	-6.07348	-0.78794	1.80002
C	-4.47274	0.31437	0.92110
O	-4.07892	1.33917	1.39529
N	-3.62864	-0.55990	0.29414
H	-3.98493	-1.46744	0.09269
C	-2.18299	-0.42303	0.43181
H	-1.91790	-0.27449	1.46901
C	-1.55588	-1.72723	-0.09305
H	-2.11705	-2.55676	0.32357
H	-1.64786	-1.78848	-1.16994
S	0.15292	-2.12175	0.39210
C	-1.65365	0.80634	-0.31064
O	-0.71420	1.41661	0.11968
N	-2.24713	1.09594	-1.48381
H	-3.13014	0.68119	-1.66789
C	-1.86694	2.27322	-2.23529
H	-0.80260	2.26270	-2.42059
H	-2.38682	2.26006	-3.18407
H	-2.11664	3.18555	-1.70523
S	1.29753	-1.42057	-1.20443
C	3.50446	1.50311	0.54723
C	2.44151	0.81090	-0.00419
C	2.60660	-0.50433	-0.41914
C	3.85221	-1.11087	-0.27927
C	4.91878	-0.42264	0.26263
C	4.74225	0.88955	0.67955
H	3.36782	2.52041	0.87316
H	1.48476	1.28516	-0.09436
O	5.80839	1.52406	1.20654
H	5.88264	-0.88295	0.37293
H	3.98496	-2.12766	-0.59928
H	5.57784	2.40412	1.47029

35

4MP beta sheet conformation

H	-5.48630	-3.06177	-0.33778
C	-4.45920	-2.92691	-0.64733
H	-4.42107	-2.73410	-1.71266
H	-3.92335	-3.84802	-0.44220
C	-3.84620	-1.81580	0.17636

O	-4.11634	-1.66080	1.33766
N	-2.96040	-1.02678	-0.47229
H	-2.71007	-1.20670	-1.41870
C	-2.25170	0.04579	0.18954
H	-2.92160	0.50348	0.90266
C	-1.03267	-0.51665	0.93736
H	-1.39049	-1.28903	1.60552
H	-0.33836	-0.96871	0.24224
S	-0.13718	0.63035	2.02995
C	-1.85518	1.03233	-0.90670
O	-1.39744	0.62508	-1.94688
N	-2.08004	2.32727	-0.65438
H	-2.35217	2.58944	0.26497
C	-1.65007	3.36471	-1.56992
H	-1.98324	3.12807	-2.56977
H	-2.09419	4.30151	-1.26221
H	-0.57144	3.46560	-1.57536
S	1.13033	1.68802	0.78056
C	4.42273	-0.67779	0.69804
C	3.44423	0.19657	1.11539
C	2.36891	0.51134	0.28525
C	2.29984	-0.05953	-0.97691
C	3.28438	-0.93610	-1.40716
C	4.34346	-1.24578	-0.57019
H	5.25421	-0.92826	1.32966
H	3.50790	0.64006	2.09170
O	5.32912	-2.09039	-0.93059
H	3.22267	-1.37329	-2.38924
H	1.47661	0.17829	-1.62560
H	5.18971	-2.41789	-1.80842

35
2MP alpha helical conformation

H	6.58129	0.10052	-0.88801
C	5.85954	-0.67656	-0.68364
H	6.05522	-1.11202	0.28999
H	5.96977	-1.45535	-1.43236
C	4.46828	-0.09017	-0.77578
O	4.22591	0.91704	-1.37277
N	3.49156	-0.79190	-0.12361
H	3.72446	-1.70799	0.18910
C	2.08385	-0.50510	-0.37315
H	1.89871	-0.43223	-1.43548
C	1.27483	-1.66982	0.22455
H	1.74431	-2.59722	-0.08529
H	1.30612	-1.64198	1.30640
S	-0.44415	-1.89412	-0.32837
C	1.65910	0.84365	0.21334
O	0.81108	1.49673	-0.32829

N	2.22482	1.19044	1.38508
H	3.05231	0.71256	1.65462
C	1.94455	2.47634	1.98880
H	0.87783	2.60256	2.10346
H	2.40739	2.50706	2.96627
H	2.32669	3.29393	1.38786
S	-1.54955	-0.87310	1.12260
C	-3.37549	2.03589	-1.03245
C	-2.42545	1.30809	-0.34978
C	-2.73815	0.05448	0.17802
C	-4.02442	-0.46367	0.01597
C	-4.98212	0.27809	-0.67006
C	-4.65515	1.51183	-1.18739
H	-3.12582	2.99751	-1.44108
H	-1.42943	1.68576	-0.23059
O	-4.40847	-1.65813	0.49028
H	-5.40362	2.07393	-1.71736
H	-5.96567	-0.13790	-0.78272
H	-3.69950	-2.06671	0.97289

35
2MP beta sheet conformation

H	-5.59011	-2.69808	-0.39288
C	-4.59438	-2.52132	-0.77361
H	-4.65009	-2.15184	-1.79066
H	-4.05953	-3.46598	-0.78081
C	-3.88142	-1.56234	0.15329
O	-4.13457	-1.49352	1.32563
N	-2.92195	-0.79966	-0.42538
H	-2.66915	-0.94060	-1.37833
C	-2.08685	0.09677	0.34244
H	-2.68314	0.49887	1.14764
C	-0.89299	-0.66589	0.93491
H	-1.28570	-1.44240	1.57888
H	-0.31999	-1.13955	0.15044
S	0.21807	0.29973	2.00730
C	-1.64017	1.19437	-0.61772
O	-1.13210	0.89620	-1.67575
N	-1.85920	2.45432	-0.24097
H	-2.21770	2.62585	0.66972
C	-1.42855	3.58081	-1.04698
H	-1.77857	3.46217	-2.06220
H	-1.85218	4.48327	-0.62894
H	-0.34915	3.66500	-1.05769
S	1.46870	1.31080	0.70153
C	4.78140	-1.00041	0.57735
C	3.80165	-0.11886	0.96983
C	2.67314	0.11193	0.17862
C	2.54248	-0.55359	-1.04083

C	3.54270	-1.44379	-1.43788
C	4.64052	-1.66325	-0.63971
H	5.64190	-1.16973	1.19780
H	3.89003	0.40904	1.90130
O	1.51288	-0.42059	-1.88255
H	5.39841	-2.35344	-0.96523
H	3.42473	-1.94598	-2.37973
H	0.86485	0.22917	-1.62310

The systems then underwent the protocol described below in the same fashion as our previous paper[3] to equilibrate the added solvent and ions and provide initial minimization of the protein environment in the solvated environment. The restraints used during equilibration were harmonic with the force constants indicated. Bonded terms for the MP sidechains were adapted by analogy from existing parameters in the ff14SB forcefield.[28] No hydrogen atoms needed to be added to the structure, as the locations were already assigned by the NMR experiments.

For these simulations, electrostatic interactions were treated with the Particle Mesh Ewald method,[29] and the van der Waals cut-off was set to 10 Å. The integration time step for all MD simulations was 1 fs, and the Langevin thermostat was used to control the temperature with a 2.0 ps^{-1} collision frequency. For the NPT ensemble, the Berendsen barostat was used.[30] Bond lengths involving hydrogen were constrained by the SETTLE algorithm[31] for the water molecules and the SHAKE algorithm[26] for the protein. AMBER20[32] was used to perform the simulations, and the ff14SB forcefield was used to treat the protein.

Minimization/Equilibration of Solvent
1. 5000 steps of minimization with the steepest descent algorithm with 500 kcal/(mol•Å^2) restraint on solute atoms.
2. 500 ps NVT equilibration at 300 K with 500 kcal/(mol•Å^2) restraint on solute atoms.
3. 1 ns NPT equilibration at 300 K and 1 atm with 200 kcal/(mol•Å^2) restraint on solute atoms.

CG - Conjugate Gradient Minimization
SD - Steepest Descent Minimization

Minimization of Solvent and Protein
4. 2000 steps SD, followed by 3000 steps CG with 100 kcal/(mol•Å^2) restraints on heavy atoms of the protein complex.
5. 2000 steps SD and 3000 steps CG with 100 kcal/(mol•Å^2) restraints on the C, C_α, and N atoms of the protein.
6. 2000 steps SD and 3000 steps CG with 50 kcal/(mol•Å^2) restraints on the C, C_α, and N atoms of the protein.
7. 2000 steps SD and 3000 steps CG with 10 kcal/(mol•Å^2) restraints on the C, C_α, and N atoms of the protein.
8. 2500 steps SD followed by 2500 steps CG with no restraints.

Equilibration of Protein/Solvent System
9. Heat system to 300 K in NPT ensemble over 360 ps in 60 ps increments, where the system is heated by 50K for 10 ps and then equilibrated at that temperature for 50 ps.
10. NPT equilibration at 300 K for 20ns.

11. NVT equilibration at 300 K for 100ns.

Production

12. NVT MD for 1 μs

A visual comparison of the NMR structures and MP-α_3C systems from the production MD trajectories is provided in Figure S17. As the forcefield parameters to describe the sulfur-aromatic carbon bonds, angles, and dihedrals were determined by analogy from existing parameters and were not widely tested, we performed additional simulations restraining the CB-S-S1-CG dihedral in both sidechains to the value found in the NMR ensemble using a 200 kcal/(mol•rad^2) force constant for the restraint. We found that the simulations with and without these restraints exhibited similar RMSDs despite sampling a much larger range of CB-S-S1-CG dihedral values compared to the NMR structures (Figures S18 and S19). The observed hydrogen-bonding interactions were also similar for the simulations with and without the dihedral restraints (Table S12).

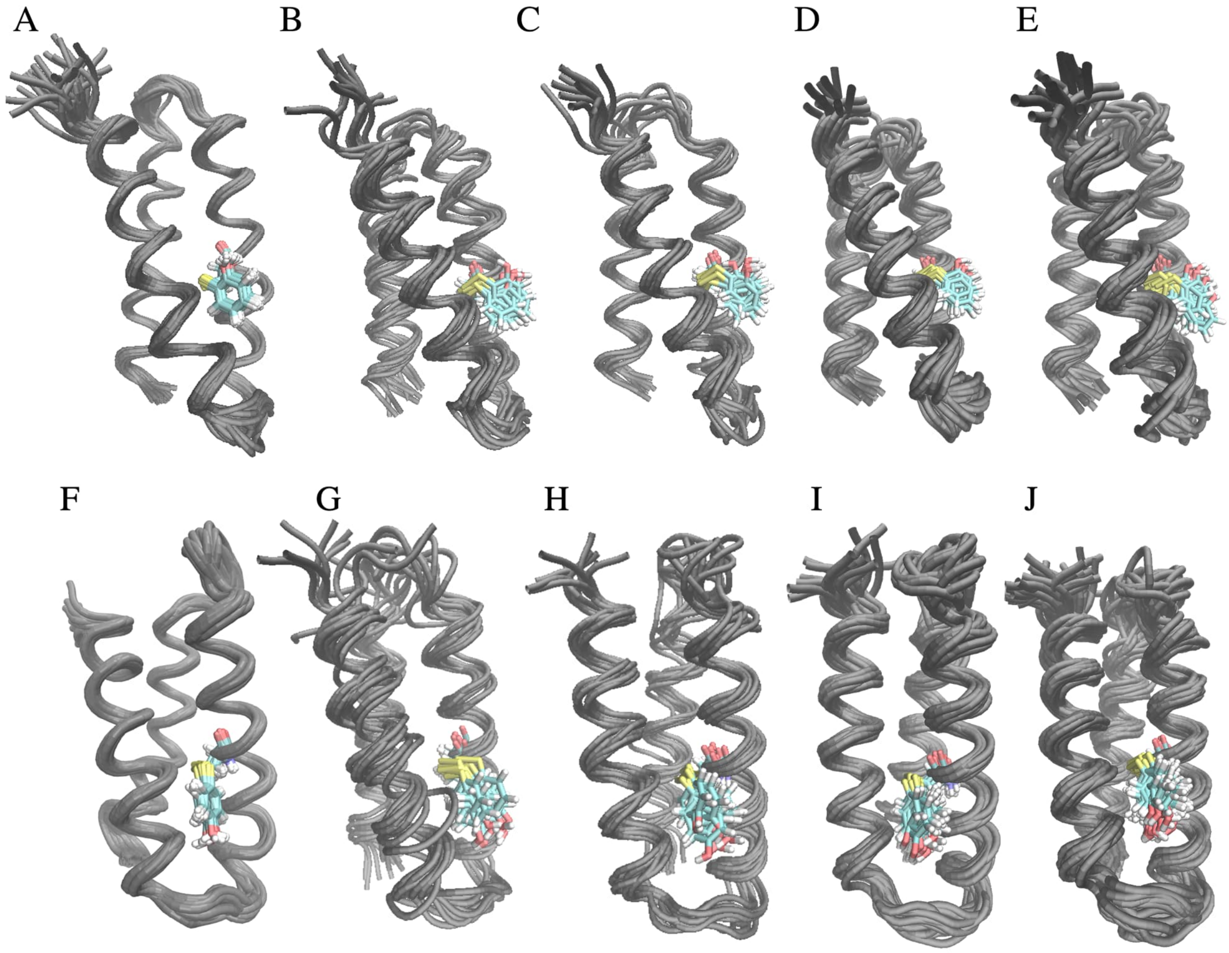

Figure S17. *NMR ensembles of 2MP-α_3C (top) and 4MP-α_3C (bottom) compared to the structures obtained from molecular dynamics.* (A,F) NMR structures; (B,G) MD conformations for trajectory 1; (C,H) MD conformations for trajectory 1 with dihedral restraints; (D,I) MD conformations for trajectory 2; (E, J) MD conformations for trajectory 2 with dihedral restraints. To obtain the MD conformations, ten conformations were abstracted from a 1 μs production trajectory at even intervals.

***Table S12**. Percentage of MD trajectories with specified hydrogen-bonding interactions involving 2MP or 4MP.*[a]

System[b]	WAT:O – MP:OH[c]	E33:OE-MP:OH	E13:OE-MP:OH[e]
2MP rest. Traj. 1	35.2%	50.5%	0.3%
2MP rest. Traj. 2	34.8%	52.4%	0.04%
2MP Traj.1	34.1%	52.1%	0.7%
2MP Traj.2	46.6%	40.6%	1.1%
4MP rest. Traj. 1	111.5%	N.D.[d]	N.D.[d]
4MP rest. Traj. 2	114.2%	<0.01%	N.D. [d]
4MP Traj.1	114.3%	0.6%	0.02%
4MP Traj.2	114.3%	0.6%	0.03%

[a]Hydrogen bonding criteria were set as a heavy atom donor-acceptor distance less than or equal to 3.0 Å and a donor-hydrogen-acceptor angle greater than or equal to 135°. Hydrogen-bonding interactions with water included 2MP or 4MP each acting as a hydrogen bond acceptor or a hydrogen bond donor, where a fraction greater than one is possible due to more than one hydrogen bond forming. Hydrogen bonds to glutamate residues can be made to either carboxylate oxygen, and the number provided is the sum of the two possibilities.

[b]The term "rest." denotes the simulations performed with a harmonic restraint on the dihedral angle CB-S-S1-CG.

[c]For previous MD simulations of the α_3Y protein, the percentage of a given trajectory with Y_{32} forming at least one hydrogen bond to a water molecule was computed to be 38.2% and 27.7% for two independent trajectories. For previous MD simulations of the α_3Y protein, the percentage of a given trajectory with Y_{32} forming at least one hydrogen bond to E13 was computed to be 24.0% and 21.6% for two independent trajectories.[3]

[d]N.D. stands for not detected.

[e]Plots of the distribution of donor-acceptor distance and donor-hydrogen-acceptor angle for the 2MP-C32 hydrogen bond with E13 or E33 for the 2MP-α_3C NMR ensembles as well as for MD trajectories are provided in Figures S20 and S21.

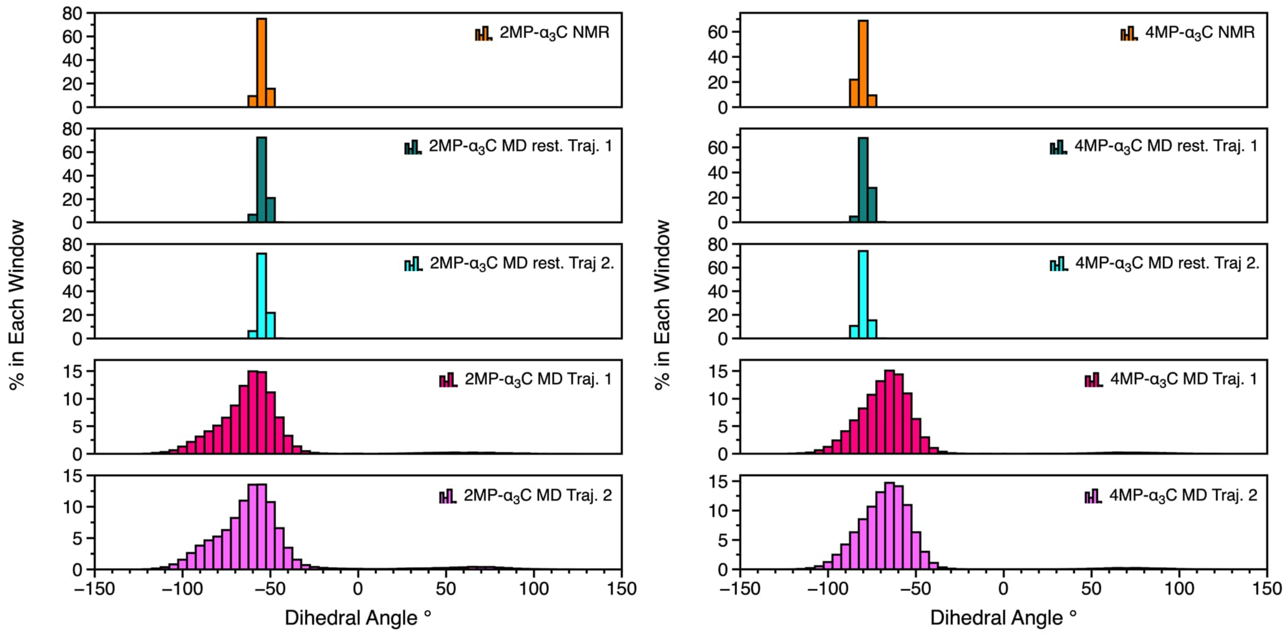

Figure S18. *Dihedral angle distributions of the CB-S-S1-CG angle for the two NMR ensembles (top row) compared to the two independent MD trajectories with a restraint on this dihedral angle (second and third rows) and the two independent MD trajectories without this restraint (fourth and fifth rows).*

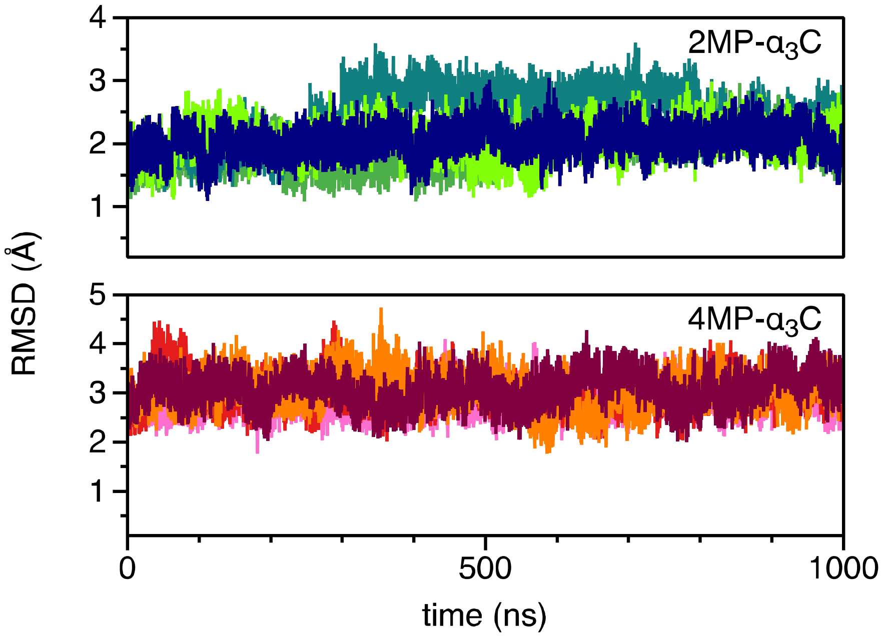

Figure S19. *RMSD of the C_α atoms relative to the starting NMR structure for the production MD simulations*. The top row are the four trajectories propagated for the 2MP-α3C system (two with the dihedral restraint and two without this restraint), and the bottom panel is for the four trajectories propagated for the 4MP-α3C system (two with the dihedral restraint and two without this restraint).

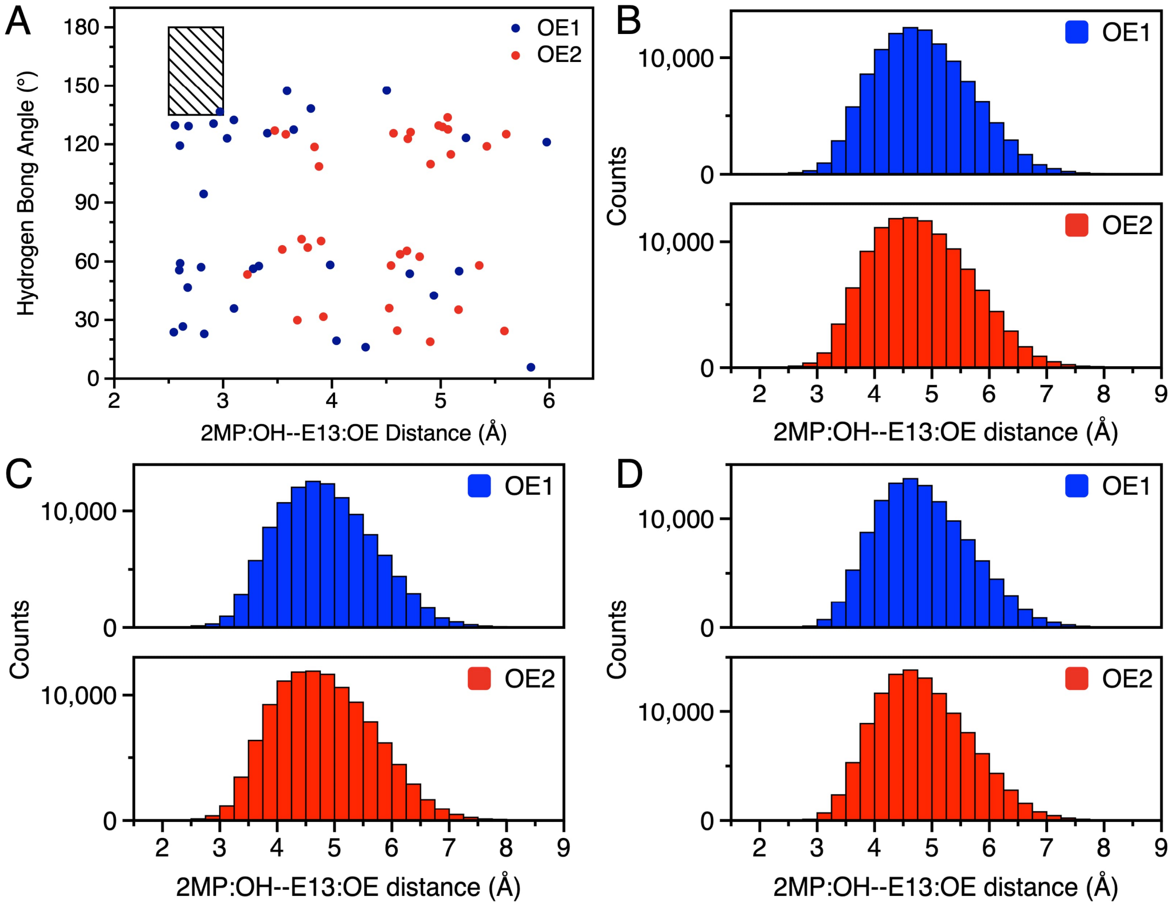

Figure S20. *Donor-acceptor distance histograms for the E13:OE-2MP:OH interaction.* (A) NMR structure ensemble; (B) 2MP Traj.1, which has no restraint on the CB-S-S1-CG dihedral. (C) 2MP Traj.2, which has no restraint on the CB-S-S1-CG dihedral. (D) 2MP rest. Traj. 2, which has a restraint on the CB-S-S1-CG dihedral. The similarity of the distribution for two independent trajectories, shown in (B) and (C), indicates convergence. In these figures, the histograms are plotted separately for each carboxylate oxygen, and the cross hatched box in panel A represents the hydrogen bond criteria used in this work. Note that the MD simulations are qualitatively consistent with the NMR structure ensemble, which does not show significant hydrogen bonding between 2MP-C32 and E13 within the hydrogen bonding criteria used in this work.

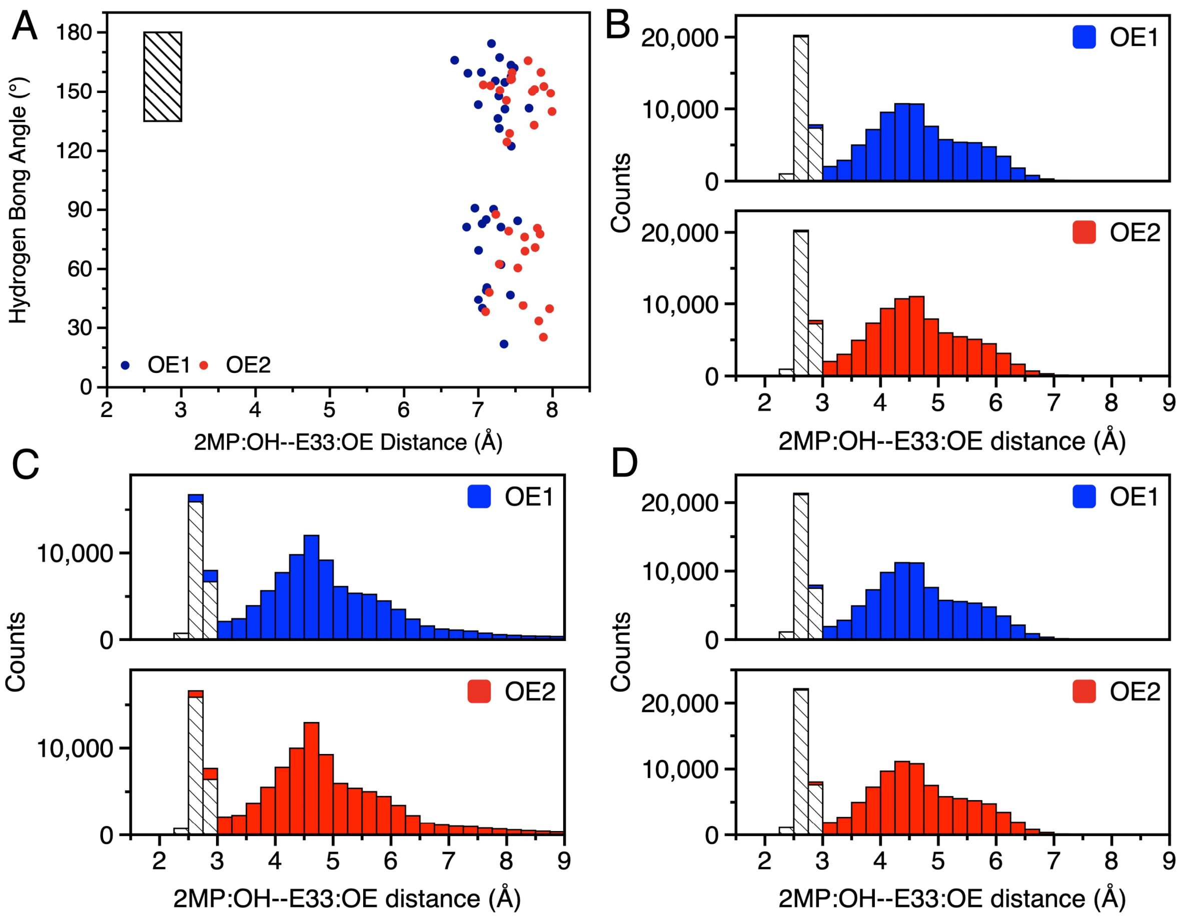

Figure S21. *Donor-acceptor distance histograms for the E33:OE-2MP:OH interaction.* (A) NMR structure ensemble; (B) 2MP Traj.1, which has no restraint on the CB-S-S1-CG dihedral. (C) 2MP Traj.2, which has no restraint on the CB-S-S1-CG dihedral. (D) 2MP rest. Traj. 2, which has a restraint on the CB-S-S1-CG dihedral. The similarity of the distribution for two independent trajectories, shown in (B) and (C), indicates convergence. In these figures, the histograms are plotted separately for each carboxylate oxygen, and the cross hatched box in panel A represents the hydrogen bond criteria used in this work. The cross-hatched bars overlayed on the histograms in panels B-D indicate the number of conformations that satisfy the distance and angle criteria for a hydrogen bond used in this work. The MD simulations show more hydrogen bonding with E33 than the NMR structure ensemble, which does not show significant hydrogen bonding between 2MP-C32 and E33.

Additional MD Simulations Probing 2MP-E13 Hydrogen Bond

Classical MD simulations with flat-welled restraint

To preserve a relatively short 2MP:OH–E13:OE distance that could facilitate hydrogen bonding, a trajectory was propagated starting from the 28th frame of the NMR structure, using an MM force field with a flat-welled restraint applied to the E13:OE1 – 2MP:OH distance. The flat region of this biasing potential was 2.5–3.0 Å (indicating that no energetic penalty was incurred in this region), with +/- 0.5 Å defining the endpoints of the parabolic region of the biasing potential. Specifically, the restraint in AMBER format was defined as R1 = 2.0 Å, R2=2.5 Å, R3 = 3.0 Å, and R4=3.5 Å. The force constants for this restraint were 20 kcal/(mol•Å^2) in the functional form $k(x-x^0)^2$. The MM force field and equilibration protocols were identical to those detailed earlier in the SI and used elsewhere in this study. Starting from the last conformation of the 1 μs trajectory, two 1 μs trajectories were propagated without the flat-welled restraint on the E13-2MP distance. The E13-2MP distance immediately became more variable, as shown in Figure S22, indicating that the hydrogen-bonding interaction between E13 and 2MP was not maintained. Thus, this molecular mechanical force field does not maintain this hydrogen-bonding interaction.

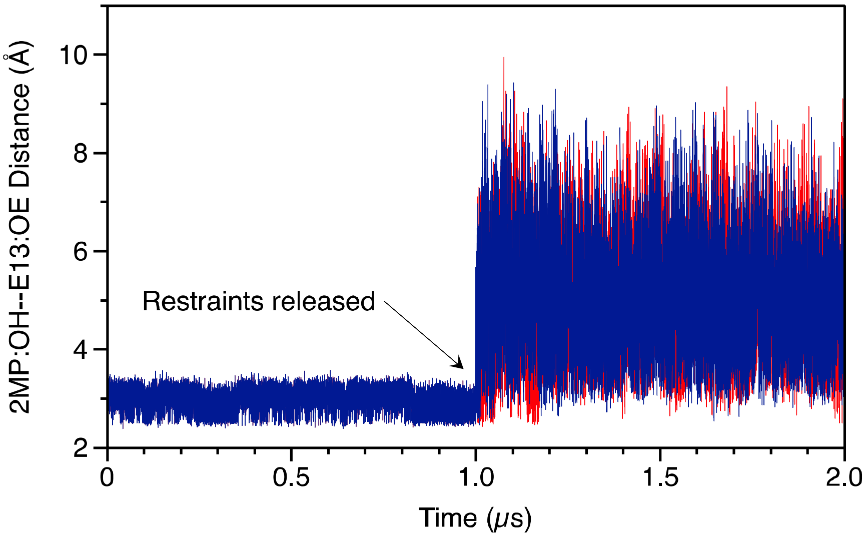

Figure S22. *Donor-acceptor distance for the E13:OE-2MP:OH interaction in an MD trajectory propagated with an MM force field and a flat-well restraint for the first half of the simulation.* The arrow indicates when the restraint was released. Two trajectories with different initial velocities were started from this configuration, as shown in dark blue and red.

QM/MM simulations

A conformation was selected from the MD trajectory described in the preceding subsection to use as a starting point for a QM/MM trajectory propagated using the AMBER/Q-Chem interface. This conformation was chosen to have a hydrogen bond between E13 and 2MP, with a donor-acceptor heavy atom distance of ~3 Å and an O-H—O angle of 156°. For the QM region, the ωB97X-D functional and 6-31G** basis set were used, as implemented in Q-Chem 5.1.[33] The QM region consisted of the E13 and 2MP sidechains, where hydrogen link atoms between the C_β and C_α atoms were added automatically by the QM/MM interface in AMBER,[34] employing the default charge redistribution scheme.[35] Only atoms within 30 Å of the QM region were allowed to move in these QM/MM simulations. The nonbonded cut-off was set to 999 Å so that no cut-off was applied. The SCF convergence tolerance was set to 10^{-6}, and the threshold for neglecting two-electron integrals was set to 10^{-14}. The QM/MM simulation was propagated for 5 ps with a 0.5 fs timestep. As shown in Figure S23, the E13:OE-2MP:OH distance decreases

to an average of ~2.6 Å, and the hydrogen bond is stable on this time scale. Note that due to the sampling limitations with QM/MM simulations, we do not expect to observe significant sidechain motions, but if the interaction were unfavorable, the donor-acceptor distance would be expected to increase, which was not observed. Thus, the hydrogen bond between E13 and 2MP appears to be stable when treating these residues quantum mechanically.

.

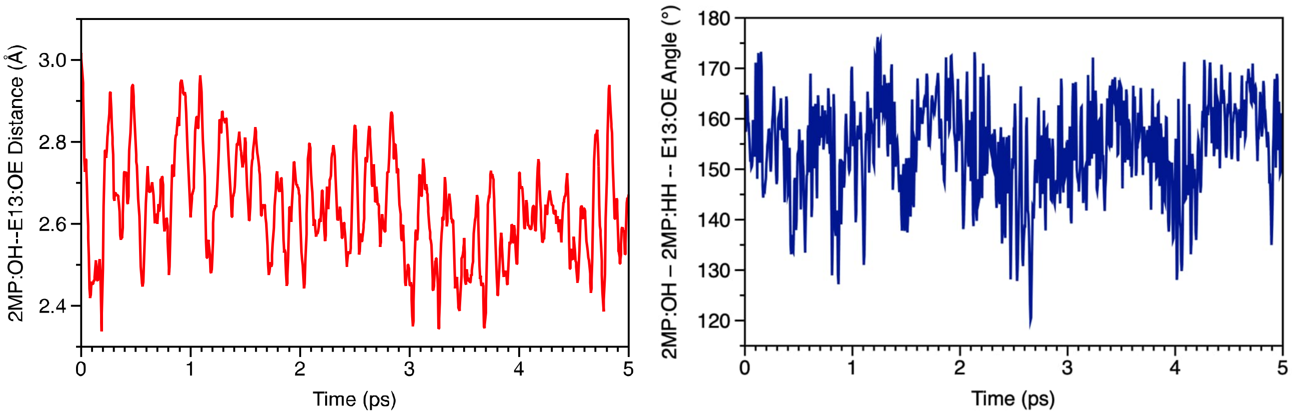

Figure S23. *Donor-acceptor distance (left) and donor-hydrogen-acceptor angle (right) for the E13:OE-2MP:OH hydrogen-bonding interaction, as obtained from a QM/MM 5 ps trajectory*. This hydrogen-bonding interaction was maintained over the 5 ps trajectory when the E13 and 2MP residues were treated quantum mechanically.

References

(1) Glover, S. D.; Jorge, C.; Liang, L.; Valentine, K. G.; Hammarstrom, L.; Tommos, C. Photochemical tyrosine oxidation in the structurally well-defined alpha3Y protein: proton-coupled electron transfer and a long-lived tyrosine radical. *J. Am. Chem. Soc.* **2014**, *136* (40), 14039-14051.
(2) Nilsen-Moe, A.; Rosichini, A.; Glover, S. D.; Hammarstrom, L. Concerted and Stepwise Proton-Coupled Electron Transfer for Tryptophan-Derivative Oxidation with Water as the Primary Proton Acceptor: Clarifying a Controversy. *J. Am. Chem. Soc.* **2022**, *144* (16), 7308-7319.
(3) Nilsen-Moe, A.; Reinhardt, C. R.; Glover, S. D.; Liang, L.; Hammes-Schiffer, S.; Hammarström, L.; Tommos, C. Proton-Coupled Electron Transfer from Tyrosine in the Interior of a de novo Protein: Mechanisms and Primary Proton Acceptor. *J. Am. Chem. Soc.* **2020**, *142* (26), 11550-11559.
(4) Frisch, M. J.; Trucks, G. W.; Schlegel, H. B.; Scuseria, G. E.; Robb, M. A.; Cheeseman, J. R.; Scalmani, G.; Barone, V.; Petersson, G. A.; Nakatsuji, H.; et al. Gaussian 16 Revision D.01. **2016**.
(5) Mulliken, R. S. Electronic Population Analysis on LCAO–MO Molecular Wave Functions. I. *J. Chem. Phys.* **1955**, *23* (10), 1833-1840.
(6) Chai, J.-D.; Head-Gordon, M. Long-range corrected hybrid density functionals with damped atom–atom dispersion corrections. *Phys. Chem. Chem. Phys.* **2008**, *10* (44), 6615-6620.
(7) Lee, C.; Yang, W.; Parr, R. G. Development of the Colle-Salvetti correlation-energy formula into a functional of the electron density. *Phys. Rev. B* **1988**, *37* (2), 785-789.
(8) Becke, A. D. Density-functional thermochemistry. III. The role of exact exchange. *J. Chem. Phys.* **1993**, *98* (7), 5648-5652.
(9) Grimme, S.; Ehrlich, S.; Goerigk, L. Effect of the damping function in dispersion corrected density functional theory. *J. Comput. Chem.* **2011**, *32* (7), 1456-1465.
(10) Zhao, Y.; Truhlar, D. G. The M06 suite of density functionals for main group thermochemistry, thermochemical kinetics, noncovalent interactions, excited states, and transition elements: two new functionals and systematic testing of four M06-class functionals and 12 other functionals. *Theor. Chem. Acc.* **2008**, *120* (1), 215-241.
(11) Hehre, W. J.; Ditchfield, R.; Pople, J. A. Self-consistent molecular orbital methods. XII. Further extensions of Gaussian—type basis sets for use in molecular orbital studies of organic molecules. *J. Chem. Phys.* **1972**, *56* (5), 2257-2261.
(12) Hariharan, P. C.; Pople, J. A. The influence of polarization functions on molecular orbital hydrogenation energies. *Theor. Chim. Acta* **1973**, *28* (3), 213-222.
(13) Clark, T.; Chandrasekhar, J.; Spitznagel, G. W.; Schleyer, P. V. R. Efficient diffuse function-augmented basis sets for anion calculations. III. The 3-21+G basis set for first-row elements, Li–F. *J. Comput. Chem.* **1983**, *4* (3), 294-301.
(14) Roos, B. O.; Taylor, P. R.; Sigbahn, P. E. M. A complete active space SCF method (CASSCF) using a density matrix formulated super-CI approach. *Chem. Phys.* **1980**, *48* (2), 157-173.
(15) Roos, B. O. The Complete Active Space Self-Consistent Field Method and its Applications in Electronic Structure Calculations. In *Adv. Chem. Phys.*, 1987; pp 399-445.
(16) Sayfutyarova, E. R.; Hammes-Schiffer, S. Constructing Molecular π-Orbital Active Spaces for Multireference Calculations of Conjugated Systems. *J. Chem. Theory Comput.* **2019**, *15* (3), 1679-1689.
(17) Jr., T. H. D. Gaussian basis sets for use in correlated molecular calculations. I. The atoms boron through neon and hydrogen. *J. Chem. Phys.* **1989**, *90* (2), 1007-1023.
(18) Woon, D. E.; Jr., T. H. D. Gaussian basis sets for use in correlated molecular calculations. III. The atoms aluminum through argon. *J. Chem. Phys.* **1993**, *98* (2), 1358-1371.
(19) Sun, Q.; Berkelbach, T. C.; Blunt, N. S.; Booth, G. H.; Guo, S.; Li, Z.; Liu, J.; McClain, J. D.; Sayfutyarova, E. R.; Sharma, S.; et al. PySCF: the Python-based simulations of chemistry framework. *WIREs: Comput. Mol. Sci.* **2018**, *8* (1).
(20) Sun, Q.; Yang, J.; Chan, G. K.-L. A general second order complete active space self-consistent-field solver for large-scale systems. *Chem. Phys. Lett.* **2017**, *683*, 291-299.
(21) Miertuš, S.; Scrocco, E.; Tomasi, J. Electrostatic interaction of a solute with a continuum. A direct utilizaion of AB initio molecular potentials for the prevision of solvent effects. *Chem. Phys.* **1981**, *55* (1), 117-129.
(22) Miertuŝ, S.; Tomasi, J. Approximate evaluations of the electrostatic free energy and internal energy changes in solution processes. *Chem. Phys.* **1982**, *65* (2), 239-245.
(23) Pascual-ahuir, J. L.; Silla, E.; Tuñon, I. GEPOL: An improved description of molecular surfaces. III. A new algorithm for the computation of a solvent-excluding surface. *J. Comput. Chem.* **1994**, *15* (10), 1127-1138.
(24) Tommos, C.; Valentine, K. G.; Martínez-Rivera, M. C.; Liang, L.; Moorman, V. R. Reversible Phenol Oxidation and Reduction in the Structurally Well-Defined 2-Mercaptophenol-α3C Protein. *Biochem.* **2013**, *52* (8), 1409-1418.
(25) Jorgensen, W. L.; Chandrasekhar, J.; Madura, J. D.; Impey, R. W.; Klein, M. L. Comparison of simple potential functions for simulating liquid water. *J. Chem. Phys.* **1983**, *79* (2), 926-935.
(26) Ryckaert, J.-P.; Ciccotti, G.; Berendsen, H. J. Numerical integration of the cartesian equations of motion of a system with constraints: molecular dynamics of n-alkanes. *J. Comput. Phys.* **1977**, *23* (3), 327-341.

(27) Cieplak, P.; Cornell, W. D.; Bayly, C.; Kollman, P. A. Application of the multimolecule and multiconformational RESP methodology to biopolymers: Charge derivation for DNA, RNA, and proteins. *J. Comput. Chem.* **1995**, *16* (11), 1357-1377.
(28) Maier, J. A.; Martinez, C.; Kasavajhala, K.; Wickstrom, L.; Hauser, K. E.; Simmerling, C. ff14SB: Improving the Accuracy of Protein Side Chain and Backbone Parameters from ff99SB. *J. Chem. Theory Comput.* **2015**, *11* (8), 3696-3713.
(29) Darden, T.; York, D.; Pedersen, L. Particle mesh Ewald: An N·log(N) method for Ewald sums in large systems. *J. Chem. Phys.* **1993**, *98* (12), 10089-10092.
(30) Berendsen, H. J. C.; Postma, J. P. M.; Gunsteren, W. F. v.; DiNola, A.; Haak, J. R. Molecular dynamics with coupling to an external bath. *J. Chem. Phys.* **1984**, *81* (8), 3684-3690.
(31) Miyamoto, S.; Kollman, P. A. SETTLE: an analytical version of the SHAKE and RATTLE algorithm for rigid water models. *J. Comput. Chem.* **1992**, *13* (8), 952-962.
(32) D. A. Case, K. Belfon, I. Y. Ben-Shalom, S. R. Brozell, D. S. Cerutti, T. E. Cheatham, III, V. W. D. Cruzeiro, T. A. Darden, R. E. Duke, G. Giambasu, M. K. Gilson, H. Gohlke, A. W. Goetz, R. Harris, S. Izadi, S. A. Izmailov, K. Kasavajhala, A. Kovalenko, R. Krasny, T. Kurtzman, T. S. Lee, S. LeGrand, P. Li, C. Lin, J. Liu, T. Luchko, R. Luo, V. Man, K. M. Merz, Y. Miao, O. Mikhailovskii, G. Monard, H. Nguyen, A. Onufriev, F. Pan, S. Pantano, R. Qi, D. R. Roe, A. Roitberg, C. Sagui, S. Schott-Verdugo, J. Shen, C. L. Simmerling, N. R. Skrynnikov, J. Smith, J. Swails, R. C. Walker, J. Wang, L. Wilson, R. M. Wolf, X. Wu, Y. Xiong, Y. Xue, D. M. York, and P. A. Kollman. 2020. AMBER 2020. University of California, San Francisco.
(33) Shao, Y.; Molnar, L. F.; Jung, Y.; Kussmann, J.; Ochsenfeld, C.; Brown, S. T.; Gilbert, A. T. B.; Slipchenko, L. V.; Levchenko, S. V.; O'Neill, D. P.; et al. Advances in methods and algorithms in a modern quantum chemistry program package. *Phys. Chem. Chem. Phys.* **2006**, *8* (27), 3172-3191.
(34) Götz, A. W.; Clark, M. A.; Walker, R. C. An extensible interface for QM/MM molecular dynamics simulations with AMBER. *J. Comput. Chem.* **2014**, *35* (2), 95-108.
(35) Walker, R. C.; Crowley, M. F.; Case, D. A. The implementation of a fast and accurate QM/MM potential method in Amber. *J. Comput. Chem.* **2008**, *29* (7), 1019-1031.
